# Predicting the Thermal Conductivity of Structural Materials Under Lead–Bismuth Corrosion Based on Machine Learning

**DOI:** 10.3390/ma19122639

**Published:** 2026-06-18

**Authors:** Xinxin Gao, Xian Zeng

**Affiliations:** 1 School of Nuclear Science and Technology, University of Science and Technology of China, Hefei 230026, China; gao_xinx@163.com; 2 China Nuclear Power Technology Research Institute Co., Ltd., Shenzhen 518031, China

**Keywords:** lead–bismuth eutectic, heat-resistant steel, corrosion, thermal conductivity, machine learning

## Abstract

316L stainless steel and T91 heat-resistant steel are key structural materials for lead-cooled fast reactors (LFRs). Lead–bismuth eutectic (LBE) corrosion induces oxide layer formation and remarkably degrades thermal conductivity, endangering reactor safety and efficiency. Systematic experimental studies on and predictive tools for the thermal conductivity of stainless steels after LBE corrosion are currently scarce. To address the lack of experimental data and predictive capabilities regarding changes in thermal conductivity following LBE corrosion, this study experimentally obtained thermal conductivity data from stainless steels after lead–bismuth corrosion and developed machine learning models to predict thermal conductivity under multi-parameter coupled LBE corrosion conditions. Three machine learning models were established using material composition and corrosion parameters as inputs. Overall, the hyperparameter-optimized Gradient Boosting Regression model showed competitive predictive performance with low overall prediction error. The model therefore provides a preliminary data-driven tool for estimating the thermal conductivity of corroded 316L stainless steel and T91 heat-resistant steel, thereby providing technical support for material selection, thermal design, and safety assessment of LFRs, with further specimen-level validation required for broader engineering application.

## 1. Introduction

Amid the escalating global energy crisis and environmental challenges, advanced nuclear energy systems have become a core strategic option for achieving “dual carbon” goals and ensuring national energy security as a clean, efficient, and low-carbon energy solution [1]. China adheres to the policy of the active, safe, and orderly development of nuclear power. By the end of 2025, the scale of China’s in-operation, approved, and under-construction nuclear power units ranks first in the world, making the establishment of a technical support system for the safe development of nuclear power a top priority [2]. As the core type of fourth-generation advanced nuclear reactors, lead-cooled fast reactors (LFRs) possess significant strategic importance for promoting the green transition of nuclear energy, owing to their inherent high safety, strong fuel breeding capacity, and capability to transmute long-lived nuclear waste [3,4].

Lead–bismuth eutectic (LBE) has become the preferred coolant and spallation target material for LFRs due to its low melting point (123.5 °C), high boiling point (1670 °C), and excellent neutronic and heat transfer properties [5,6]. 316L austenitic stainless steel and T91 ferritic/martensitic stainless steel are the mainstream structural material candidates for LFR core components, fuel cladding, and heat transfer pipelines, owing to their excellent mechanical properties, good processability, and favourable neutronic economy. Their long-term service performance in the LBE environment directly determines the operational safety and service life of the reactor [7,8]. Under actual operating conditions of LFRs (temperature: 300–600 °C, dissolved oxygen concentration: 10^−9^–10^−6^ wt.%, flow rate: 0–5 m/s), 316L and T91 stainless steels undergo corrosion reactions with liquid LBE, forming oxide layers mainly composed of Fe3O4 and Fe–Cr spinel on their surfaces. The thickness, density, and phase composition of these oxide layers evolve dynamically with corrosion parameters [9].

As the interfacial transition layer between LBE and the material matrix, the thermal conductivity of the oxide layer is only 1/5–1/3 that of the matrix material. Additionally, its porosity and interfacial bonding state introduce additional thermal resistance, directly reducing the overall thermal conductivity of the material [10]. Thermal conductivity is a core parameter that characterizes a material’s heat transfer capacity. Its degradation significantly impairs the efficiency of heat transfer from the core to the coolant. Moreover, it may trigger safety hazards, such as local overheating of the fuel cladding and heat exchange imbalance, while also reducing the operational economy of the reactor [11].

The impact of the oxide layer on the thermal conductivity of structural materials is of great importance in engineering. However, existing research exhibits a notable imbalance. The vast majority of studies focus on the corrosion behaviour of stainless steels in LBE environments and the formation mechanisms of oxide layers, exploring how operating parameters influence corrosion rate, oxide layer phase, and morphology [12,13]. In contrast, research on changes in the thermal conductivity of corroded materials remains scarce, with almost no relevant experimental data available.

In the heat transfer design and safety assessment of LFRs, the thermal conductivity of structural materials is currently assumed to be constant, without considering the dynamic influence of oxide layer evolution. This assumption easily leads to deviations in core temperature prediction. If an excessively large conservative calculation margin is adopted, the economy of the reactor will be reduced [14]. Moreover, systematic experimental investigations and predictive methodologies concerning the thermal conductivity of stainless steels following lead–bismuth corrosion remain highly limited. The OECD/NEA Handbook on Lead–Bismuth Eutectic Alloy and Lead Properties [6], together with relevant International Atomic Energy Agency reports [11], merely indicates that the oxide layer growth rate should be appropriately controlled and that the degradation in material thermal conductivity should remain within 25% throughout the operational lifetime (i.e., a design lifespan of 40–60 years). However, no detailed quantitative evaluation regarding the influence of corrosion on thermal conductivity degradation has been reported.

As a core tool for transforming materials science from an empirical trial-and-error paradigm to a data-driven approach, machine learning has been widely applied in fields such as material property prediction and microstructure analysis [15,16]. However, it has not yet been applied to predict the thermal conductivity of corroded LFR structural materials. The implicit correlations among corrosion parameters, microstructure characteristics, and thermal conductivity have not been effectively explored, and traditional theoretical models struggle to adapt to the complex corrosion–heat conduction coupling relationship in LBE environments [17].

To fill the gap in experimental data and predictive tools related to the thermal conductivity changes in stainless steels after LBE corrosion, this study takes 316L and T91 stainless steels as the research objects. By simulating the actual operating conditions of LFRs, static LBE corrosion experiments with multi-parameter coupling were designed and conducted to systematically measure the thermal conductivity of materials under different corrosion conditions and to construct a measured thermal conductivity database. Drawing on the concept of “structural descriptor + deep learning”, material composition, corrosion conditions, and test conditions were selected as input features to develop multiple machine learning models for thermal conductivity prediction. The optimal model was identified through comprehensive evaluation using multiple indicators. The influence of each feature on thermal conductivity was analyzed, ultimately leading to the development of a practical prediction tool for the thermal conductivity of structural materials under lead–bismuth corrosion conditions, thereby providing data support and technical means for the design and safety assessment of LFRs.

## 2. Corrosion Experiments in an LBE Environment

### 2.1. Experimental Materials and Corrosion-Conditions Design

**Figure 1:**
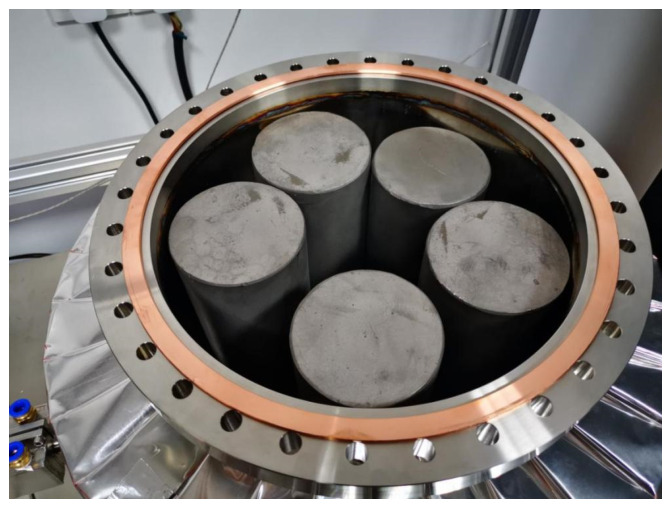
Lead–bismuth ingot.

This study selected 316L and T91 stainless steels as the research objects. Based on the actual operating conditions of LFRs, 97 sets of static LBE corrosion experiments were designed and conducted, covering potential operating conditions in LFRs, including temperatures of 400–600 °C, corrosion times of 1000–9000 h, 1 × 10^−8^ wt.%~saturated oxygen concentration, and Bi contents of 50–60 wt.%. Thermal conductivity measurements were performed at 350 °C, 400 °C, 450 °C, and 500 °C, which covers the typical operating temperature window of LFRs, yielding 388 sets of experimental data for machine learning model training. Additionally, 11 sets of supplementary validation experiments were conducted within the aforementioned range of corrosion conditions, and thermal conductivity measurements were performed at the same four temperatures to obtain data. Figure 1 shows a physical photograph of the LBE alloy ingot. The LBE ingots used in the experiments were smelted from Pb and Bi according to specific mass percentages (Pb50Bi50, Pb47Bi53, Pb44.5Bi55.5, Pb40Bi60).

**Figure 2:**
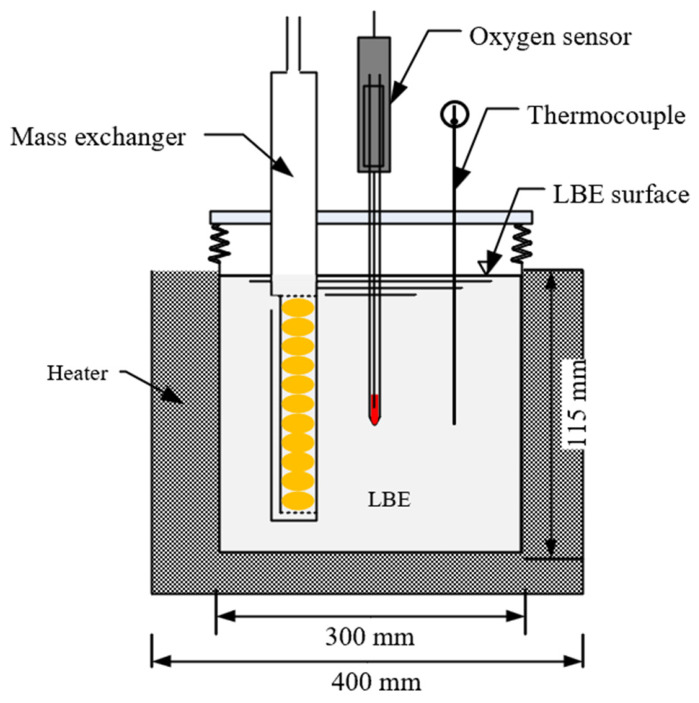
Static lead–bismuth alloy corrosion experimental setup (the yellow section represents lead oxide filled in the solid-state oxygen control system, while the red section denotes the reference electrode inside the sensor).

The configuration of the lead–bismuth alloy corrosion experiment is illustrated in Figure 2. Its core components include an oxygen sensor, a temperature-measuring thermocouple, gas inlet and outlet pipelines, an experimental tank, and an automatic oxygen control system, which can accurately regulate temperature and oxygen concentration during the corrosion process. The experimental tank is fabricated from 316L stainless steel. To prevent the liquid LBE alloy from corroding the tank and affecting experimental accuracy, an alumina crucible is incorporated as a protective lining inside the tank.

**Figure 3:**
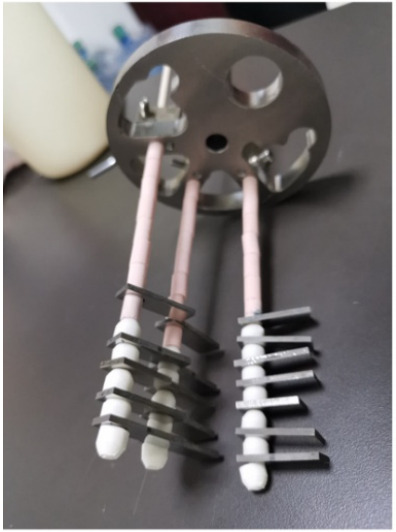
Sample suspension.

The sample suspension and fixation scheme is as Figure 3. To minimize interference from the sample holder on corrosion, the holder is constructed from an alumina ceramic rod with a diameter of Φ3.5 mm and a length of 150 mm. Various samples are connected in series into a column through pre-drilled holes on one side of the samples, with Φ5 mm alumina round tubes separating adjacent samples. After assembly, the ceramic rod carrying the samples is fixed to a dedicated sample suspension frame using screws.

The specific experimental procedure is as follows: first, a block-shaped, solid LBE alloy is placed into the alumina crucible inside the tank and heated to 200 °C until it is completely melted; subsequently, the suspension frame with fixed samples is positioned directly above the tank, and the height of the ceramic rod is precisely adjusted to ensure that all series-connected samples are fully immersed in the liquid LBE; after sealing the tank lid with bolts, the temperature is further increased to 600 °C and maintained at a constant level, after which the corrosion experiment timing is initiated.

This study employed an La_1−x_Sr_x_Co_1−__Ɣ_Fe_Ɣ_O_3−δ_ (LSCF)/air reference-type oxygen sensor to achieve real-time online monitoring of oxygen concentration in LBE, and precise regulation was accomplished using a PID closed-loop control algorithm. As a perovskite-structured mixed ionic–electronic conductor, LSCF exhibits both high oxygen ionic conductivity and electronic conductivity, as well as rapid oxygen adsorption, dissociation, and diffusion capabilities at high temperatures, thereby providing the oxygen sensor with advantages such as fast response, good stability, and a wide applicable temperature range [18]. Oxygen content measurement is achieved by detecting the potential response induced by changes in oxygen partial pressure. When the oxygen concentration exceeds the set threshold, the control system automatically adjusts the injection flow rates of Ar + air and Ar + 5%H_2_ mixed gases to achieve dynamic regulation of oxygen concentration.

### 2.2. Sample Treatment and Thermal Conductivity Testing

After the samples are corroded under the specified conditions for a defined period, the experimental tank is cooled to room temperature, and the suspension frame along with the samples is removed. Each sample is sectioned using wire electrical discharge machining, which offers an extremely small heat-affected zone and high machining precision, thereby effectively avoiding phase transformation or detachment of the corrosion layer microstructure caused by thermal effects during the cutting process. The corroded samples are processed into 10 × 10 × 2 mm square sheets using a wire cutting machine for subsequent thermal conductivity measurement.

For samples designated for thermal conductivity testing, residual liquid LBE alloy adhering to the surface must first be removed. Chemical cleaning is employed for this purpose, using a mixed solution of ethanol, hydrogen peroxide, and acetic acid with a volume ratio of 1:1:1 as the cleaning agent. This ternary mixed system exhibits a synergistic cleaning effect: acetic acid, as a weakly acidic medium, chemically reacts with the LBE alloy to form soluble products such as lead acetate; hydrogen peroxide acts as an oxidizing agent, accelerating the dissolution of the LBE alloy; ethanol serves as a co-solvent, improving the compatibility of each component and facilitating the prompt dissolution of reaction products to prevent their secondary deposition on the sample surface. The cleaning process is conducted at room temperature by fully immersing the samples in the mixed solution. After cleaning, an ultrasonic cleaner is used to remove residual solution from the surface, followed by drying to complete surface pretreatment.

The thermal conductivity of the samples is characterized using the laser flash method. The sample surface is first pretreated by carbon sputtering to reduce laser reflectivity and improve energy absorption efficiency, and then a laser flash thermal conductivity meter is employed for testing. To reduce test variability and ensure data repeatability and reliability, each sample is measured at three points, with each point tested repeatedly three times. The arithmetic mean of the three repeated measurements is taken as the thermal conductivity characterization result of the sample.

Prior to fabricating the corroded specimens, five 316L samples were extracted from different locations within the same steel batch, and their thermal conductivities were measured at 350 °C, 400 °C, 450 °C, and 500 °C. The corresponding pre-corrosion thermal conductivity values of 316L stainless steel are summarized in Table 1. Within the temperature interval of 350 °C to 500 °C, the measured thermal conductivity values varied from 20.707 W/(m·K) to 21.551 W/(m·K), which falls within the characteristic thermal conductivity range reported for austenitic stainless steels, i.e., 15 W/(m·K) to 25 W/(m·K).

Following the same procedure, five T91 samples were extracted from different locations within the same steel batch, and their thermal conductivities were measured at 350 °C, 400 °C, 450 °C, and 500 °C. The corresponding pre-corrosion thermal conductivity values of T91 steel are presented in Table 2.

In this study, a Netzsch LFA 467 instrument (supplied by NETZSCH Analyzing & Testing, Selb, Germany) was employed to perform thermal conductivity measurements. Relevant studies [19] have shown that the laser flash technique demonstrates high precision and reliability over a broad temperature range and under various experimental conditions, with repeatability and reproducibility of approximately ±1% and an accuracy ranging from 3% to 5% based on reference-material validation. Furthermore, the instrument software automatically provides the statistical precision (repeatability) together with the combined uncertainty associated with the measurement results. Statistical evaluation of the generated outputs indicates a maximum uncertainty of 0.4% and a maximum standard deviation of 4.8%.

## 3. Machine Learning Model Construction and Evaluation Methods

### 3.1. Establishment of Thermal Conductivity Database

In this study, owing to limitations in time, resources and operational safety, it is difficult to acquire a large quantity of fully independent specimens and test conditions in this work. Accordingly, a selective sampling strategy was implemented, with parameters chosen to cover typical and accident conditions of lead-cooled fast reactors. Experiments were conducted on T91 and 316L stainless steels under the corrosion environments listed in Table 3. This table lists the specific corrosion temperature, corrosion time, oxygen concentration, and Bi content applied in the experiments. A total of 108 static LBE corrosion experiments were conducted. From these, 97 experiments (388 thermal conductivity measurements) were randomly selected as the training set. In addition, 44 measurement data obtained under 11 corrosion conditions were adopted as Supplementary Materials.

### 3.2. Feature Selection

In the machine learning model constructed in this study, eight parameters belonging to three categories—namely, material properties, external corrosion conditions, and test conditions—were selected as independent input features, with material thermal conductivity serving as the target feature. The material properties correspond to the core chemical compositions of 316L and T91 (Ni, Fe, Cr); the external corrosion conditions include corrosion temperature, corrosion time, dissolved oxygen concentration, and Bi content; the test condition corresponds to the thermal conductivity measurement temperature.

The model adopts a grouped five-fold cross-validation strategy in which the corrosion conditions serve as the grouping criterion: datasets sharing identical material type, composition, corrosion temperature, corrosion duration, oxygen concentration, and Bi content are assigned to the same group, and all measurement-temperature data points within each group are allocated exclusively to either the training fold or the validation fold.

The influence mechanisms of each input feature on thermal conductivity are as follows: (1) The chemical composition of the material directly determines the crystal structure, phase composition, and interatomic binding energy, thereby serving as the fundamental factor influencing thermal conductivity. (2) The measurement temperature affects the phonon scattering efficiency within the material, acting as a key environmental parameter in thermal conductivity testing. (3) Corrosion temperature and time collectively determine the thickness, density, and stability of the oxide layer, and variations in the microstructure of the oxide layer significantly hinder the heat conduction process. (4) Oxygen concentration and Bi content influence the corrosion reaction rate and product type, thereby indirectly altering the material’s heat conduction performance.

Prior to model development, the input data were numerically encoded, examined for missing values, and subjected to sensitivity processing for exact duplicate records. The random seed applied during model training was fixed at 42.

### 3.3. Selection of Machine Learning Models

This study selected three classical ensemble learning regression models to construct the thermal conductivity prediction model: RFR, GBR, and XGB. The core principles of each model are as follows:

(1) RFR: This is a non-parametric tree-based model founded on the Bagging framework. It generates multiple mutually independent training subsets through bootstrap sampling, trains a decision tree base learner on each subset, randomly selects a subset of features for optimal partitioning during node splitting, and ultimately predicts continuous dependent variables by averaging the outputs of multiple decision trees. Its core concept is a variance reduction strategy: reducing the overall variance of the model through the independence and randomness of base learners, thereby mitigating the overfitting risk of a single decision tree [20].
(2) GBR: This is an additive model under the Boosting framework. It iteratively generates weak learners (typically shallow decision trees) in a forward stepwise manner. Each iteration fits the negative gradient (i.e., residual approximation) of the previous model output, minimizes a predefined loss function (such as squared loss or absolute loss) through gradient descent, and ultimately obtains regression predictions through the weighted summation of all weak learners. In terms of the training mechanism, RFR is based on the Bagging framework, constructing multiple mutually independent decision trees through parallel training, with final predictions obtained by voting or averaging; in contrast, GBR adopts a sequential training mechanism, in which each decision tree is generated iteratively. The core objective of each subsequent decision tree is to compensate for the prediction bias of the preceding model, thereby achieving progressive error correction. Specifically, the GBR model aims to reduce prediction bias as its primary optimization objective, continuously correcting prediction errors through multiple iterations and progressively approximating the distribution characteristics of real data, thereby improving regression accuracy and generalization performance [21].
(3) XGB: This is a regularization-enhanced extension of the traditional GBR framework. Its core innovations include refined optimization of the loss function and the integration of multiple regularization mechanisms. It approximates the loss function using a second-order Taylor expansion, incorporates multiple regularization techniques such as L1 regularization (Lasso), L2 regularization (Ridge), learning rate decay, leaf node weight constraints, column sampling, and row sampling, and employs approximate greedy algorithms and sparse-aware optimization to enhance computational efficiency. This approach improves model generalization and stability while maintaining strong fitting capability. In essence, XGB is a regularized GBR tree model that balances prediction accuracy, computational efficiency, and overfitting control. In recent years, the XGBoost model has additionally been employed as an efficient surrogate model for CFD simulations, enabling rapid predictions with high computational accuracy [22].

To ensure the reliability of the results, it is essential to evaluate the predictive performance of all regression models to identify the optimal model. A single evaluation metric frequently reflects only one aspect of model performance; therefore, a combination of multiple metrics is recommended to comprehensively characterize the model behaviour across different dimensions [23]. For regression tasks, mean absolute error (MAE) and root mean square error (RMSE) are common evaluation metrics. Additionally, the coefficient of determination ($R^{2}$) is employed to assess the proportion of variance in the target variable explained by the regression model. After selecting an appropriate model based on these three metrics, this study further calculated the mean absolute percentage error (MAPE) of the optimal model to verify its predictive accuracy. Since MAPE reflects the average deviation between predicted and true values without being affected by data dimensionality, it enables direct comparison of predicted thermal conductivity results. In this study, these four metrics are used to evaluate model performance, and their combined use provides a more comprehensive assessment. Their mathematical expressions are presented in the following equations.

The formula for $R^{2}$ is as follows:

(1) $$R^{2}=1-\frac{\sum_{i=1}^{n}(y_{i}-{\hat{y}}_{i}{)}^{2}}{\sum_{i=1}^{n}(y_{i}-\bar{y}{)}^{2}},$$

where $y_{i}$ is the true value of the *i*-th sample;$\;{\hat{y}}_{i}$ is the predicted value of the i-th sample;$\;\bar{y}$ is the average of all sample true values; and n: is the number of samples.

The RMSE is as follows:

(2) $$RMSE=\sqrt{\frac{1}{n}\sum_{i=1}^{n}(y_{i}-{\hat{y}}_{i}{)}^{2}}.$$

The MAE is as follows:

(3) $$MAE=\frac{1}{n}\sum_{i=1}^{n}|y_{i}-{\hat{y}}_{i}|.$$

The MAPE is as follows:

(4) $$MAPE=\frac{1}{n}\times \sum_{i=1}^{n}\frac{\left|y_{i}-{\hat{y}}_{i}\right|}{y_{i}}\times 100\%.$$

The characteristics of the above four indicators are compared in Table 4.

## 4. Results and Discussion

### 4.1. Feature Importance Analysis

**Figure 4:**
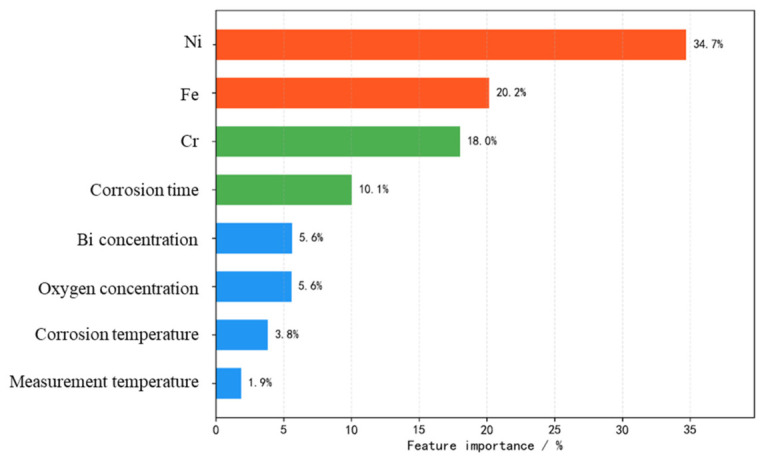
Contribution of input parameters to the prediction results.

Figure 4 shows the contribution of eight input parameters to the prediction results. The feature importance is calculated based on the built-in feature_importances_ attribute of the GBR model, which averages the reduction in impurity (variance) contributed by each feature across all trees.

The combined importance of Ni, Fe, and Cr accounts for 72.9%, indicating that alloy composition has the largest predictive contribution in this two-material dataset. This result should be interpreted as reflecting the compositional difference between 316L and T91 rather than proving an independent causal effect of any single element. Corrosion time is the next most important factor in the built-in importance analysis, accounting for 10.1%. As corrosion time increases, an oxide layer or corrosion products form on the material surface.

### 4.2. Comparison of Different Machine Learning Model Performances

The three models (RFR, GBR, XGB) were first compared under the original row-wise data split and conventional 5-fold cross-validation, using R^2^, RMSE, and MAE as screening metrics.

All data processing, model development, hyperparameter optimization, and statistical analyses were performed using Python 3.12. Data manipulation and numerical computations were conducted using the pandas and numpy libraries. Machine learning models, data standardization, grouped cross-validation, grid-search optimization, and performance evaluation metrics were implemented using scikit-learn. The XGBoost model was established using the xgboost library. Excel files were imported using openpyxl, whereas figures were generated using matplotlib. The random seed was fixed at 42 to enhance reproducibility.

**Figure 5:**
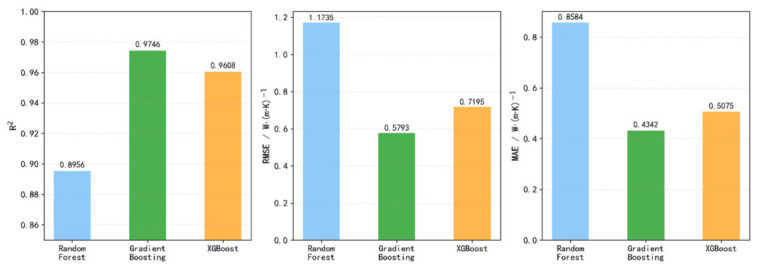
Coefficient of determination ($R^{2}$), root mean square error (RMSE), and mean absolute error (MAE) of the Random Forest Regression, Gradient Boosting Regression, and eXtreme Gradient Boosting Regression models.

The results are shown in Figure 5. Under the original row-wise model-screening setting, the GBR model achieved the best apparent performance, followed by the XGB model, while the RFR model exhibited the lowest performance.

The R^2^ value of the GBR model is 0.9746, the R^2^ value of the XGB model is 0.9608, and the R^2^ value of the RFR model is 0.8956. These results indicate that the Boosting-based models achieved higher apparent fitting performance than RFR under the original row-wise split. However, because measurements from the same corrosion condition may appear in both training and test subsets under row-wise splitting, these metrics are used as model-screening results and not as sole evidence of leakage-controlled generalization.

The RMSE value of the GBR model is 0.5793 W/(m·K), which is the lowest among the three models under the original row-wise split. The RMSE value of the XGB model is 0.7195 W/(m·K), ranking second, whereas the RMSE value of the RFR model is 1.1735 W/(m·K). These differences support selecting GBR for subsequent analysis, while the leakage-controlled grouped validation in Section 4.6 is used to evaluate generalization more strictly.

The MAE value of the GBR model is 0.4342 W/(m·K), the MAE value of the XGB model is 0.5075 W/(m·K), and the MAE value of the RFR model is 0.8584 W/(m·K). The MAE results are consistent with the RMSE results and support GBR as the preferred candidate model for the subsequent grouped-validation and supplementary-validation analyses.

These initial results are consistent with the tendency of Boosting models to capture nonlinear trends in the present dataset. However, after controlling for corrosion-condition leakage, the advantage of the tuned GBR model over simple baseline models was limited, as discussed in Section 4.6.

### 4.3. Analysis of GBR Model Fitting Accuracy

**Figure 6:**
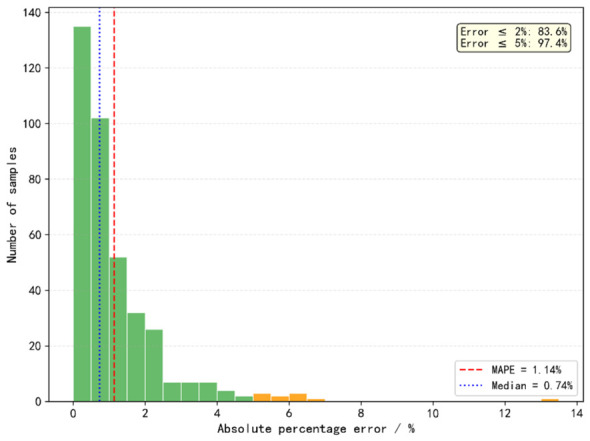
Mean absolute percentage error of the Gradient Boosting Regression model.

**Figure 7:**
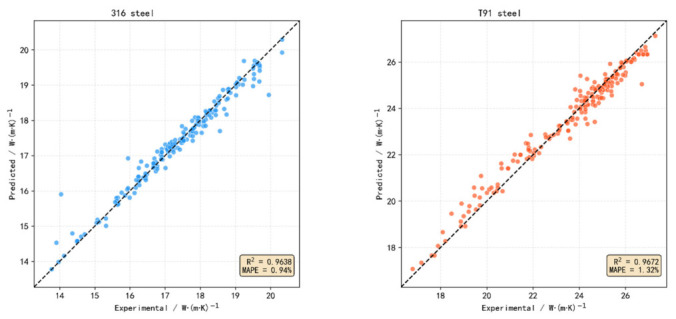
Mean absolute percentage error of T91 and 316L in the Gradient Boosting Regression model.

The fitting accuracy of the GBR model was further evaluated using MAPE. The overall MAPE of the GBR model on the 388 modelling-data records (Table S1) is 1.14% (Figure 6), indicating low apparent prediction error within the original modelling data. In Figure 6, Green bars represent samples with absolute percentage error ≤ 5%, and orange bars denote samples with absolute percentage error above 5%. The red dashed line marks the MAPE value of 1.14%, and the blue dotted line indicates the median error of 0.74%. To further analyze model performance for different materials, evaluation metrics for 316L and T91 were analyzed separately. As shown in Figure 7, for 316L, R^2^ = 0.9638 and MAPE = 0.94%, whereas for T91, R^2^ = 0.9672 and MAPE = 1.32%. These results indicate good modelling-data fitting for both steels. Blue dots denote data points of predicted versus experimental thermal conductivity for 316 steel, while orange dots represent data points for T91 steel. The black dashed diagonal line is the ideal reference line where predicted values are equal to experimental values.

**Figure 8:**
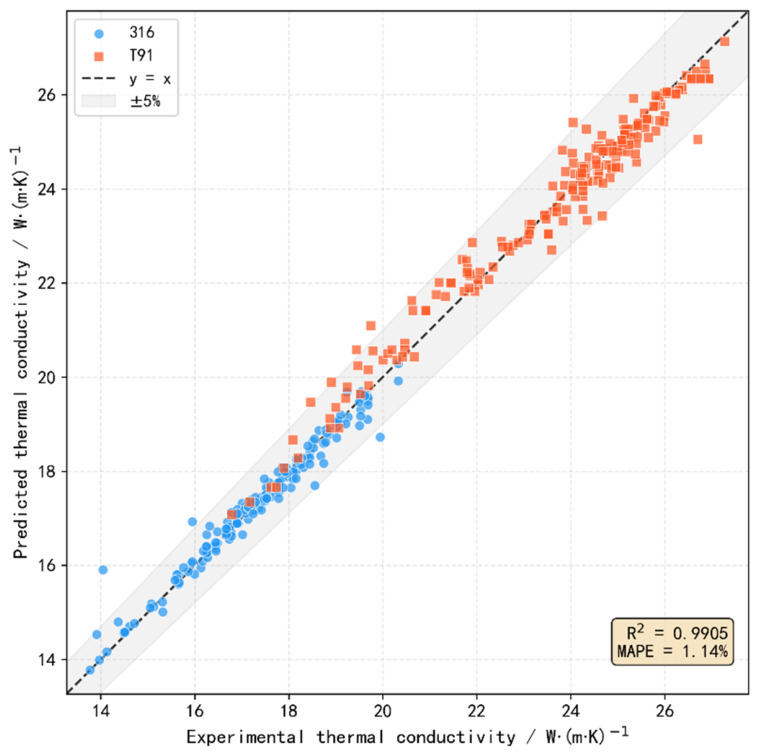
Comparison scatter plot of predicted values and measured values in the Gradient Boosting Regression model.

The comparison scatter plot of predicted and measured values (Figure 8) shows that most sample points of 316L and T91 are closely distributed along the y = x ideal reference line and within the ±5% error tolerance band, without evident systematic deviations. The overall $R^{2}$ of the model reaches 0.9905, indicating that it explains more than 99% of the data variation. Among these, T91 samples are primarily distributed in the higher thermal conductivity range of >24 W/(m·K), whereas 316L samples are concentrated in the lower thermal conductivity range of 14–20 W/(m·K). The model maintains stable predictive performance across different thermal conductivity ranges.

**Figure 9:**
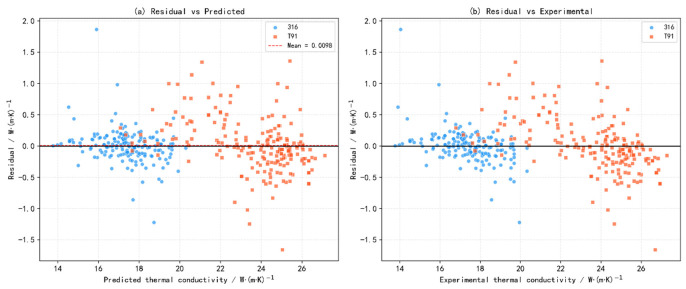
Residual distribution of thermal conductivity prediction for 316L and T91 by the Gradient Boosting Regression model: (**a**) residual vs. predicted values; (**b**) residual vs. measured values.

Residual analysis was conducted for the GBR model, and scatter plots of residuals versus predicted values and residuals versus measured values were generated (Figure 9). The results show that the residuals of 316L and T91 fluctuate randomly around the zero line, with mean residuals close to zero and no systematic deviation as predicted or measured values increase. This indicates that prediction errors primarily arise from experimental random noise rather than model specification bias, further confirming the reliability of the GBR model.

### 4.4. Verification of the GBR Model Generalization Ability

**Figure 10:**
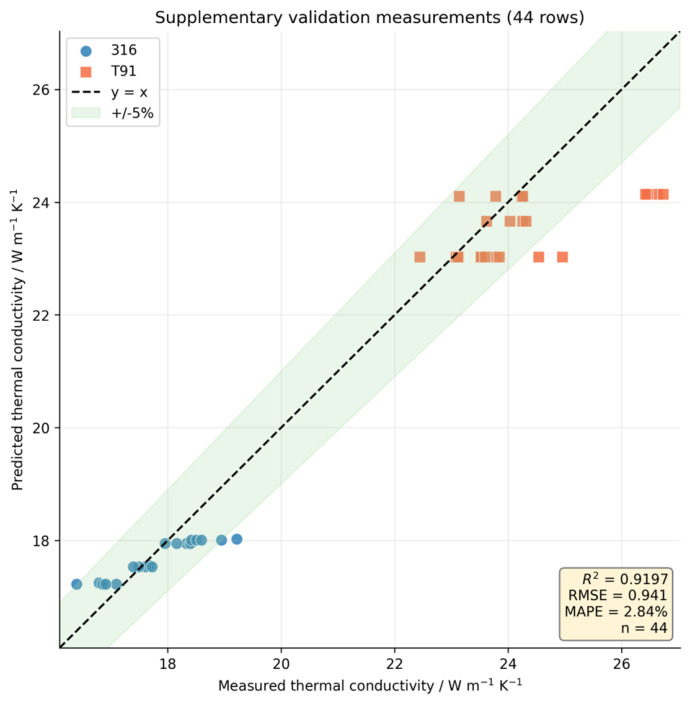
Predicted values vs. measured values of the supplementary validation measurements.

The predictive performance of the GBR model was further evaluated using 44 supplementary validation measurements from 11 corrosion-condition groups. The comparison between predicted and measured values indicates that most sample points are distributed near the y = x reference line and within the ±5% error tolerance band. As shown in Figure 10, the model achieved R^2^ = 0.9197, MAPE = 2.84%, RMSE = 0.9413 W/(m·K), and MAE = 0.6429 W/(m·K). These results indicate a low overall prediction error in the Supplementary Materials. Because specimen-level source independence could not be fully verified, this result is reported as supplementary validation rather than strict external validation.

**Figure 11:**
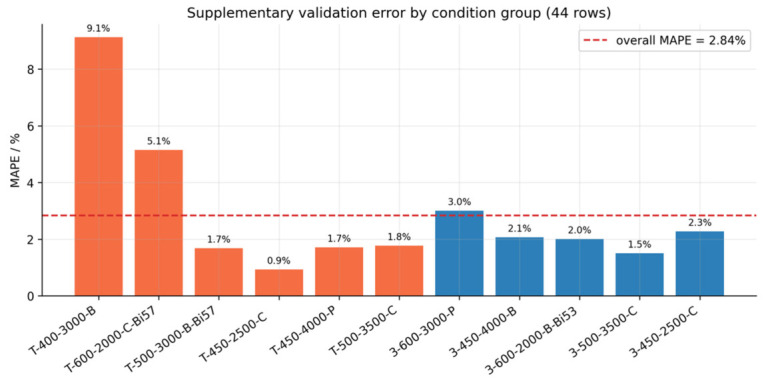
Bar chart of the mean absolute percentage error (MAPE) by supplementary validation condition group.

The bar chart of MAPE grouped by supplementary validation condition (Figure 11) shows that mean prediction errors vary across different materials and operating conditions. The group-level MAPE values range from 0.93% to 9.13%, with an overall MAPE of 2.84%. The highest group-level MAPE occurs for T-400-3000-B (9.13%), followed by T-600-2000-C-Bi57 (5.15%), whereas most other groups are below or close to 3%. These results indicate that the model maintains a low average supplementary-validation error, but its prediction error can increase for sparsely represented or boundary corrosion conditions.

Table 5 compares the performance metrics of the GBR model on the modelling data and the Supplementary Materials. The supplementary validation metrics are lower than the modelling-data fitting metrics but remain within a low-error range. Moreover, 96.4% of the modelling-data records and 81.8% of the supplementary validation records exhibit prediction errors below 5%. Compared with machine learning models for material thermal conductivity reported in the literature, the present model shows competitive prediction accuracy: Sun and Hu [24] reported a thermal conductivity prediction model for crystalline materials with MAE = 0.39 W/(m·K) and $R^{2}$ = 0.96 on the training set; Wang et al. [25] applied the XGB model to predict thermal conductivity, achieving MAE = 2.13 W/(m·K) and $R^{2}$ = 0.90 on the test set; in contrast, the GBR model in this study achieves MAE = 0.2371 W/(m·K) and $R^{2}$ = 0.9905 on the training set, and MAE = 0.7596 W/(m·K) and $R^{2}$ = 0.9175 on the validation set.

### 4.5. Hyperparameter Optimization of the GBR Model

The primary hyperparameters of the fixed-parameter Gradient Boosting Regression model were specified as n_estimators = 100, max_depth = 5, and learning_rate = 0.1. To further assess the influence of hyperparameter configurations on model performance, grid-search optimization based on corrosion-condition grouping was employed for hyperparameter tuning. The corresponding search ranges were defined as n_estimators = [50, 100, 200], learning_rate = [0.03, 0.1], max_depth = [2, 3, 5], and min_samples_leaf = [1, 2]. Grouped cross-validation within the training folds was subsequently applied, with RMSE adopted as the evaluation metric for parameter selection. The optimal hyperparameter combination obtained through grid-search optimization for the Gradient Boosting Regression model was determined as n_estimators = 100, learning_rate = 0.03, max_depth = 2, and min_samples_leaf = 1.

**Figure 12:**
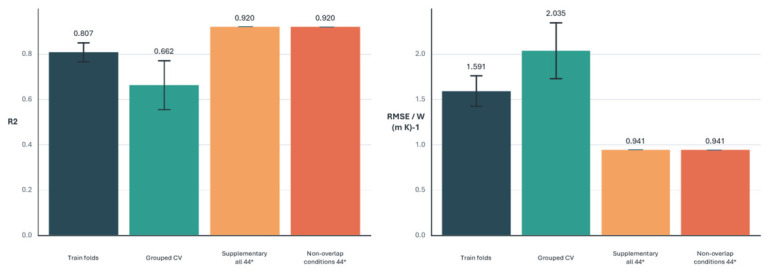
Performance of the tuned Gradient Boosting Regression model across training folds, corrosion-condition grouped cross-validation, and supplementary validation measurements. * Supplementary measurements are reported separately; source independence cannot be fully verified.

Figure 12 compares the performance of the tuned Gradient Boosting Regression model across the training folds, corrosion-condition-grouped cross-validation, and supplementary validation measurements. The grouped cross-validation results exhibited lower predictive capability than those obtained for the training folds, with $R^{2}$ decreasing from 0.807 to 0.662 and RMSE increasing from 1.591 to 2.035 W/(m·K). These results indicate that the model performance declined when corrosion-condition leakage was effectively controlled. The 44 supplementary validation measurements yielded an $R^{2}$ value of 0.920 and an RMSE of 0.941 W/(m·K), demonstrating a relatively low overall prediction error.

### 4.6. Comparison with Simple Baseline Models

In this study, several simple baseline models were additionally established to compare the proposed model with linear regression, empirical regression, and a composition-only prediction model. All models were evaluated under the same corrosion-condition-grouped five-fold cross-validation framework.

**Figure 13:**
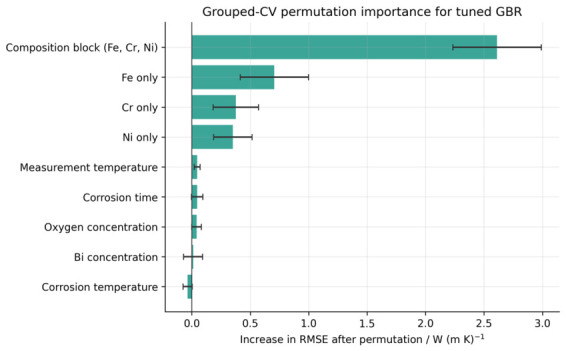
Permutation importance of the tuned Gradient Boosting Regression model evaluated on grouped cross-validation folds. Importance is reported as the increase in RMSE after permuting each feature block or feature.

Figure 13 presents the results of the permutation-importance analysis conducted on the tuned Gradient Boosting Regression model under the corrosion-condition-grouped five-fold cross-validation framework. The y-axis lists the input features or feature blocks, whereas the x-axis represents the increase in RMSE (in W/(m·K)) following random permutation of the feature values, thereby quantifying the contribution of each feature to the predictive performance of the model. The “composition block” (Fe, Cr, Ni) was permuted as an integrated feature block to evaluate the overall influence of alloy composition. The remaining evaluated features included measurement temperature, corrosion duration, oxygen concentration, Bi concentration, and corrosion temperature.

Permuting the composition block leads to the largest increase in RMSE, indicating that alloy composition is the most influential predictor within the present two-material dataset. The individual Fe, Cr, and Ni variables also show larger RMSE increases than the corrosion and test-condition variables. Among the non-composition variables, the RMSE increases are small and close to each other.

Combined with the previous feature importance analysis, corrosion time remains more influential than corrosion temperature, oxygen concentration, and Bi concentration, thereby identifying corrosion duration as the dominant operational factor.

**Figure 14:**
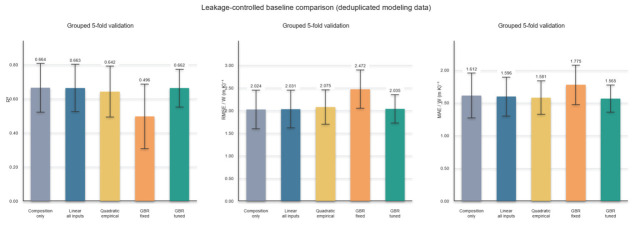
Leakage-controlled model comparison under corrosion-condition grouped 5-fold cross-validation.

The grouped validation results (Figure 14) demonstrated that the tuned Gradient Boosting Regression model achieved an RMSE of 2.035 ± 0.315 W/(m·K), whereas the composition-only linear regression model yielded 2.024 ± 0.425 W/(m·K), the full-feature linear regression model produced 2.031 ± 0.418 W/(m·K), and the quadratic empirical baseline model generated 2.075 ± 0.382 W/(m·K). Although the tuned Gradient Boosting Regression model did not achieve a statistically significant lower mean RMSE compared to simple linear baselines under leakage-controlled grouped cross-validation, it exhibited a substantially smaller standard deviation, indicating superior cross-fold stability and robustness. This consistency is particularly valuable for engineering applications where reliable predictions across diverse corrosion scenarios are critical. Moreover, the GBR model outperformed the quadratic empirical baseline in both mean and standard deviation, further supporting its practical utility. Nevertheless, compared with the fixed-parameter Gradient Boosting model, the tuned Gradient Boosting Regression model exhibited improved predictive performance.

## 5. Conclusions

Targeting the technical demand for predicting the thermal conductivity of LFR structural materials in LBE corrosion environments, this study selected 316L and T91 stainless steels as the research objects, conducted static LBE corrosion experiments with multi-parameter coupling, constructed a measured thermal conductivity database, selected material composition, corrosion conditions, and test conditions as input features, developed and optimized multiple machine learning models for thermal conductivity prediction, and analyzed model performance and feature effects under both original and leakage-controlled validation settings. The main conclusions are as follows:

There were 108 sets of static LBE corrosion experiments that were designed and conducted, covering corrosion temperatures of 400–600 °C, corrosion times of 1000–9000 h, oxygen concentrations of 1 × 10^−8^ wt.% saturated oxygen concentration, and Bi contents of 50–60%. A total of 388 sets of training data and 44 Supplementary Materials (Table S2) were obtained, establishing a measured thermal conductivity database of 316L and T91 stainless steels under LBE corrosion conditions, thereby addressing the lack of experimental data in this field.

In the current dataset containing only two material grades, namely, 316L and T91 stainless steels, the importance of Fe, Cr, and Ni primarily reflects the overall compositional differences between the two materials and their predictive contributions, with a total contribution of 72.9%. Corrosion time is the most critical operating parameter (10.1%). Bi content and oxygen concentration indirectly affect thermal conductivity by modulating the corrosion reaction, while the influences of corrosion temperature and measurement temperature are relatively limited.

Among the three constructed machine learning models (RFR, GBR, XGB), the GBR model demonstrates the best predictive performance, offering high precision and low bias. However, the apparent performance of the model decreased slightly after leakage-controlled grouped validation, indicating that row-wise splitting can overestimate generalization when measurements from the same corrosion condition are separated across folds.

Three types of baseline models were additionally established, including a composition-only linear regression model, a full-feature linear regression model, and a predefined quadratic empirical regression model. Under the strict grouped-evaluation framework, the tuned Gradient Boosting Regression model did not achieve a statistically significant lower mean RMSE than the simple linear baselines, but exhibited superior cross-fold stability (smaller standard deviation) and outperformed the quadratic empirical baseline. For the 44 supplementary validation measurements, the model achieved an *R*^2^ of 0.9197, an RMSE of 0.9413 W/(m·K), an MAE of 0.6429 W/(m·K), and a MAPE of 2.84%, demonstrating a relatively low overall prediction error.

Among the complex models, the GBR model can provide rapid data-driven estimates of the thermal conductivity of 316L stainless steel and T91 heat-resistant steel in LBE corrosion environments, thereby providing a preliminary predictive tool for material selection, heat transfer system design, and operational safety assessment of LFRs, as well as offering a methodological reference for predicting material thermophysical properties under other corrosion environments. Nevertheless, given the current dataset size, additional independent datasets are required to further validate the potential advantages associated with more sophisticated models. Looking ahead, future work may integrate machine learning models with LFR thermal-hydraulic design software to develop an engineering application module, optimize material composition and corrosion operating parameters based on feature importance analysis to propose thermal conductivity optimization strategies, and conduct long-term service validation experiments to further improve the generalization and reliability of the model, thereby providing more comprehensive technical support for the engineering application of LFRs.

## Figures and Tables

**Table 1:** Thermal conductivity of uncorroded 316L.

Temperature (°C)	Thermal Conductivity (W/(m·K))
350	20.707
400	21.407
450	21.496
500	21.551

**Table 2:** Thermal conductivity of uncorroded T91.

Temperature (°C)	Thermal Conductivity (W/(m·K))
350	27.092
400	27.062
450	26.623
500	25.101

**Table 3:** Corrosion environment conditions used in machine learning.

Conditions	Specific Parameters	Specific Parameters	Specific Parameters	Specific Parameters	Specific Parameters	Specific Parameters	Specific Parameters
Corrosion temperature (°C)	350	400	450	500	550	600	/
Corrosion time (h)	1000	2000	3000	4000	5000	7000	9000
Oxygen concentration (wt.%)	3.45 × 10^−5^	1.16 × 10^−4^	3.31 × 10^−4^	8.23 × 10^−4^	1.83 × 10^−3^	1 × 10^−6^	1 × 10^−8^
Bi content (%)	50	53	55.5	57	60	/	/

**Table 4:** Comparison of model evaluation indicators.

Indicators	Core of Formula	Unit	Characteristics
$R^{2}$	Residual/total variation	Dimensionless	Overall degree of fit, closer to 1 is better
RMSE	Mean of squared errors	Same as original data	Sensitive to large errors
MAE	Mean of absolute errors	Same as original data	Intuitive, treats large and small errors equally
MAPE	Mean of error percentages	Percentage	Relative indicator, facilitates cross-dimensional comparison

**Table 5:** Comparison of the validation and training results of the Gradient Boosting Regression model.

Indicators	Validation Results	Training Results
$R^{2}$	0.9197	0.9905
RMSE	0.9413	0.3589
MAE	0.6429	0.2371
MAPE	2.84%	1.14%
Data volume	44	388
Error < 2%	18/44 (40.9%)	321/388 (82.7%)
Error < 5%	36/44 (81.8%)	374/388 (96.4%)

## Data Availability

The original contributions presented in this study are included in the article/Supplementary Material. Further inquiries can be directed to the corresponding author.

## Supplementary Materials

The following supporting information can be downloaded at: https://www.mdpi.com/article/10.3390/ma19122639/s1, Table S1: Raw thermal conductivity measurement dataset for model training (388 groups of data). Table S2: Supplementary independent validation dataset under different LBE corrosion conditions (44 groups of data).

## References

[1] Yang Y., Zhou P., Ye G., Yang H., Hu Y., Song Y. (2025). Advanced nuclear energy development scenario study. At. Energy Sci. Technol..

[2] Hu C. (2025). China nuclear energy technology innovation development report: Global nuclear energy capacity is expected to exceed 1.1 billion kW by 2050. China Nucl. Ind..

[3] Wu Y., Wang M., Huang Q., Zhao Z., Hu L., Song Y., Jiang J., Li C., Long P. (2015). Development status and prospects of lead-based reactors. Nucl. Sci. Eng..

[4] Generation IV International Forum Technology Roadmap Update for Generation IV Nuclear Energy Systems, 2014. https://www.gen-4.org/resources/reports/technology-roadmap-update-generation-iv-nuclear-energy-systems-gif-2014.

[5] Zhu Z., Zhang Q., Tan J., Wu X., Ma H., Zhang Z., Ren Q., Han E.-H., Wang X. (2022). Corrosion behavior of T91 steel in liquid lead-bismuth eutectic at 550 °C: Effects of exposure time and dissolved oxygen concentration. Corros. Sci..

[6] OECD Nuclear Energy Agency (2015). Handbook on Lead-Bismuth Eutectic Alloy and Lead Properties, Materials Compatibility, Thermalhydraulics and Technologies.

[7] Tan J., Zhang X., Xue B., Zhang Z., Wu X. (2025). Research progress on environmental compatibility evaluation in liquid lead bismuth eutectic of stainless steels used in lead-cooled fast reactor. Nucl. Tech..

[8] Kurata Y., Futakawa M., Saito S. (2008). Corrosion behavior of steels in liquid lead–bismuth with low oxygen concentrations. J. Nucl. Mater..

[9] Wang J., Li H., Li H., Zheng W., Lv C., Ma Y., Ren S., Bao S., He Y., Yang J. (2023). Research progress on compatibility of ferritic/martensitic steel and austenitic stainless steel in static lead-bismuth eutectic environments. High Power Laser Part. Beams.

[10] Liu Y., Du J., Zhou Z., Tian C., Liu Z., Zhang Y., Shi K., Chen Z. (2024). Thermally-induced fracture in the oxide scale of T91 ferritic/martensitic steel after exposure to oxygen-saturated liquid lead–bismuth eutectic. Eng. Fract. Mech..

[11] International Atomic Energy Agency (2002). Comparative Assessment of Thermophysical and Thermohydraulic Characteristics of Lead, Lead-Bismuth and Sodium Coolants for Fast Reactors.

[12] Benamati G., Fazio C., Piankova H., Rusanov A. (2002). Temperature effect on the corrosion mechanism of austenitic and martensitic steels in lead–bismuth. J. Nucl. Mater..

[13] Yeliseyeva O., Tsisar V., Benamati G. (2008). Influence of temperature on the interaction mode of T91 and AISI 316L steels with Pb–Bi melt saturated by oxygen. Corros. Sci..

[14] Ji X., Liu X., Zhang T., He H. (2024). Numerical investigation on the Multi-Physics coupling characteristics of Lead-Bismuth reactor fuel assembly based on oxidation corrosion behaviors. Nucl. Eng. Des..

[15] Jiang X., Fu H., Bai Y., Jiang L., Zhang H., Wang W., Yun P., He J., Xue D., Lookman T. (2025). Interpretable machine learning applications: A promising prospect of AI for materials. Adv. Funct. Mater..

[16] Chen L., Tran H., Batra R., Kim C., Ramprasad R. (2019). Machine learning models for the lattice thermal conductivity prediction of inorganic materials. Comput. Mater. Sci..

[17] Liu L., Jia Z., Yuan J., Bao R., Luo H., Gu H., Zhao P. (2025). A study of corrosion products deposition behavior in a lead-bismuth-cooled wire-wrapped rod bundle based on flow-thermophysical field parameters. Energy.

[18] Liang R., Zhu H., Yang L., Qi M., Li X., Wang Y., Sheng Z., Tu X., Xu G., Li B. (2023). Performance of potentiometric oxygen sensors with LSCF electrode in lead–bismuth eutectic loop. Ann. Nucl. Energy.

[19] Blumm J. (2025). The Laser Flash Technique: A Widespread Technology for Measurement of the Thermal Diffusivity of Solids and Liquids. Int. J. Thermophys..

[20] Breiman L. (2001). Random forests. Mach. Learn..

[21] Friedman J.H. (2001). Greedy function approximation: A gradient boosting machine. Ann. Stat..

[22] Milićević A. (2026). Numerical Simulation-Driven Machine Learning and Particle Swarm Optimization of Burner Fuel Distribution for Cleaner Combustion in a Thermal Power Plant. Eng. Appl. Artif. Intell..

[23] Khoshvaght H., Permala R.R., Razmjou A., Khiadani M. (2025). A critical review on selecting performance evaluation metrics for supervised machine learning models in wastewater quality prediction. J. Environ. Chem. Eng..

[24] Sun Y., Hu W. (2021). Novel machine learning framework for thermal conductivity prediction by crystal graph convolution embedded ensemble. SmartMat.

[25] Wang X., Zeng S., Wang Z., Ni J. (2020). Identification of crystalline materials with ultra-low thermal conductivity based on machine learning study. J. Phys. Chem. C.

