# Supplementary material for: Predicting the Thermal Conductivity of Structural Materials Under Lead–Bismuth Corrosion Based on Machine Learning
Source: Materials (Basel). 2026 Jun 18;19(12):2639. doi: 10.3390/ma19122639 (PMC13302900; doi:10.3390/ma19122639)
Supplement: Supplementary file 1 [file materials-19-02639-s001.zip › materials-4322665-supplementary.pdf]

Supplementary Materials

Table S1

Training Data

Fe (wt.%)	Cr (wt.%)	Ni (wt.%)	Corrosion temperature (°C)	Corrosion time (h)	Oxygen concentration (wt.%)	Bi concentration (%)	Measurement temperature (°C)	Measured thermal conductivity	Predicted thermal conductivity	Absolute deviation	Percentage deviation (%)
0.9102	0.0574	0.0014	500	4000	1.00E−08	55.5	350	24.8701	24.4889	0.3813	1.53
0.9102	0.0574	0.0014	500	4000	1.00E−08	55.5	400	24.7688	24.5069	0.2618	1.06
0.9102	0.0574	0.0014	500	4000	1.00E−08	55.5	450	26.7077	25.0480	1.6597	6.21
0.9102	0.0574	0.0014	500	4000	1.00E−08	55.5	500	25.4036	24.5706	0.8331	3.28
0.9102	0.0574	0.0014	450	2000	1.00E−06	55.5	350	23.6209	23.5095	0.1113	0.47
0.9102	0.0574	0.0014	450	2000	1.00E−06	55.5	400	23.9085	23.5581	0.3504	1.47
0.9102	0.0574	0.0014	450	2000	1.00E−06	55.5	450	24.8406	24.2344	0.6062	2.44
0.9102	0.0574	0.0014	450	2000	1.00E−06	55.5	500	24.2393	24.1413	0.0979	0.40
0.9102	0.0574	0.0014	600	3000	1.00E−06	50	350	23.8705	24.0729	0.2025	0.85
0.9102	0.0574	0.0014	600	3000	1.00E−06	50	400	24.0645	24.0729	0.0084	0.04

Fe (wt.%)	Cr (wt.%)	Ni (wt.%)	Corrosion temperature (°C)	Corrosion time (h)	Oxygen concentration (wt.%)	Bi concentration (%)	Measurement temperature (°C)	Measured thermal conductivity	Predicted thermal conductivity	Absolute deviation	Percentage deviation (%)
0.9102	0.0574	0.0014	600	3000	1.00E−06	50	450	25.0296	24.4430	0.5866	2.34
0.9102	0.0574	0.0014	600	3000	1.00E−06	50	500	24.5817	24.3238	0.2579	1.05
0.9102	0.0574	0.0014	600	3000	1.00E−08	53	350	22.3450	22.3381	0.0070	0.03
0.9102	0.0574	0.0014	600	3000	1.00E−08	53	400	21.8632	22.1488	0.2856	1.31
0.9102	0.0574	0.0014	600	3000	1.00E−08	53	450	22.7003	22.6813	0.0190	0.08
0.9102	0.0574	0.0014	600	3000	1.00E−08	53	500	21.6915	22.4970	0.8054	3.71
0.9102	0.0574	0.0014	500	5000	1.00E−08	55.5	350	25.2746	25.2657	0.0089	0.04
0.9102	0.0574	0.0014	500	5000	1.00E−08	55.5	400	25.2442	25.2680	0.0238	0.09
0.9102	0.0574	0.0014	500	5000	1.00E−08	55.5	450	24.0506	25.4093	1.3587	5.65
0.9102	0.0574	0.0014	500	5000	1.00E−08	55.5	500	24.5932	24.9180	0.3247	1.32
0.9102	0.0574	0.0014	600	8500	1.00E−08	55.5	350	25.1349	25.2500	0.1151	0.46
0.9102	0.0574	0.0014	600	8500	1.00E−08	55.5	400	25.1755	25.2704	0.0949	0.38
0.9102	0.0574	0.0014	600	8500	1.00E−08	55.5	450	24.3325	25.2704	0.9379	3.85
0.9102	0.0574	0.0014	600	8500	1.00E−08	55.5	500	24.9584	24.9096	0.0488	0.20

Fe (wt.%)	Cr (wt.%)	Ni (wt.%)	Corrosion temperature (°C)	Corrosion time (h)	Oxygen concentration (wt.%)	Bi concentration (%)	Measurement temperature (°C)	Measured thermal conductivity	Predicted thermal conductivity	Absolute deviation	Percentage deviation (%)
0.9102	0.0574	0.0014	500	7000	1.00E−06	55.5	350	25.0870	24.8307	0.2563	1.02
0.9102	0.0574	0.0014	500	7000	1.00E−06	55.5	400	24.9594	24.6873	0.2721	1.09
0.9102	0.0574	0.0014	500	7000	1.00E−06	55.5	450	24.0560	24.5476	0.4916	2.04
0.9102	0.0574	0.0014	500	7000	1.00E−06	55.5	500	24.6020	24.3129	0.2891	1.18
0.9102	0.0574	0.0014	450	1500	1.00E−06	55.5	350	23.0744	22.9152	0.1592	0.69
0.9102	0.0574	0.0014	450	1500	1.00E−06	55.5	400	22.7762	22.8063	0.0301	0.13
0.9102	0.0574	0.0014	450	1500	1.00E−06	55.5	450	21.9052	22.8562	0.9510	4.34
0.9102	0.0574	0.0014	450	1500	1.00E−06	55.5	500	21.7806	22.4770	0.6964	3.20
0.9102	0.0574	0.0014	550	1500	1.00E−06	55.5	350	20.2944	20.3571	0.0627	0.31
0.9102	0.0574	0.0014	550	1500	1.00E−06	55.5	400	20.0023	20.3571	0.3547	1.77
0.9102	0.0574	0.0014	550	1500	1.00E−06	55.5	450	19.2116	19.5499	0.3383	1.76
0.9102	0.0574	0.0014	550	1500	1.00E−06	55.5	500	18.8718	19.1227	0.2510	1.33
0.9102	0.0574	0.0014	450	1000	1.00E−08	55.5	350	20.4697	20.7196	0.2500	1.22
0.9102	0.0574	0.0014	450	1000	1.00E−08	55.5	400	20.1064	20.5053	0.3989	1.98

Fe (wt.%)	Cr (wt.%)	Ni (wt.%)	Corrosion temperature (°C)	Corrosion time (h)	Oxygen concentration (wt.%)	Bi concentration (%)	Measurement temperature (°C)	Measured thermal conductivity	Predicted thermal conductivity	Absolute deviation	Percentage deviation (%)
0.9102	0.0574	0.0014	450	1000	1.00E−08	55.5	450	19.2469	19.7888	0.5419	2.82
0.9102	0.0574	0.0014	450	1000	1.00E−08	55.5	500	18.9061	19.8927	0.9866	5.22
0.9102	0.0574	0.0014	600	3000	1.00E−08	57	350	19.5267	19.6379	0.1112	0.57
0.9102	0.0574	0.0014	600	3000	1.00E−08	57	400	18.9979	19.3548	0.3569	1.88
0.9102	0.0574	0.0014	600	3000	1.00E−08	57	450	18.4667	19.4632	0.9965	5.40
0.9102	0.0574	0.0014	600	3000	1.00E−08	57	500	18.0874	18.6666	0.5792	3.20
0.9102	0.0574	0.0014	450	4000	1.00E−06	55.5	350	22.8930	22.8623	0.0308	0.13
0.9102	0.0574	0.0014	450	4000	1.00E−06	55.5	400	22.5302	22.8812	0.3510	1.56
0.9102	0.0574	0.0014	450	4000	1.00E−06	55.5	450	22.0723	22.2294	0.1571	0.71
0.9102	0.0574	0.0014	450	4000	1.00E−06	55.5	500	22.0317	21.9604	0.0713	0.32
0.9102	0.0574	0.0014	500	1000	1.00E−08	55.5	350	26.0115	25.5541	0.4573	1.76
0.9102	0.0574	0.0014	500	1000	1.00E−08	55.5	400	25.6661	25.4571	0.2090	0.81
0.9102	0.0574	0.0014	500	1000	1.00E−08	55.5	450	24.9195	24.7637	0.1558	0.63
0.9102	0.0574	0.0014	500	1000	1.00E−08	55.5	500	24.7695	24.8676	0.0981	0.40

Fe (wt.%)	Cr (wt.%)	Ni (wt.%)	Corrosion temperature (°C)	Corrosion time (h)	Oxygen concentration (wt.%)	Bi concentration (%)	Measurement temperature (°C)	Measured thermal conductivity	Predicted thermal conductivity	Absolute deviation	Percentage deviation (%)
0.9102	0.0574	0.0014	500	1000	1.00E−06	55.5	350	24.6046	24.4763	0.1283	0.52
0.9102	0.0574	0.0014	500	1000	1.00E−06	55.5	400	24.3236	24.4763	0.1526	0.63
0.9102	0.0574	0.0014	500	1000	1.00E−06	55.5	450	24.2930	24.3632	0.4700	1.97
0.9102	0.0574	0.0014	500	1000	1.00E−06	55.5	500	23.8243	24.8219	0.9976	4.19
0.9102	0.0574	0.0014	400	1500	1.00E−06	55.5	350	24.6707	23.4227	1.2480	5.06
0.9102	0.0574	0.0014	400	1500	1.00E−06	55.5	400	24.3518	23.3270	1.0248	4.21
0.9102	0.0574	0.0014	400	1500	1.00E−06	55.5	450	23.4400	23.4226	0.0174	0.07
0.9102	0.0574	0.0014	400	1500	1.00E−06	55.5	500	23.4495	23.4415	0.0081	0.03
0.9102	0.0574	0.0014	350	3000	3.45E−05	55.5	350	25.5263	25.3801	0.1462	0.57
0.9102	0.0574	0.0014	350	3000	3.45E−05	55.5	400	25.3404	25.1570	0.1835	0.72
0.9102	0.0574	0.0014	350	3000	3.45E−05	55.5	450	24.3895	24.6698	0.2802	1.15
0.9102	0.0574	0.0014	350	3000	3.45E−05	55.5	500	24.8763	24.4303	0.4460	1.79
0.9102	0.0574	0.0014	450	3000	3.31E−04	55.5	350	26.3848	26.1017	0.2831	1.07
0.9102	0.0574	0.0014	450	3000	3.31E−04	55.5	400	25.9184	25.7879	0.1305	0.50

Fe (wt.%)	Cr (wt.%)	Ni (wt.%)	Corrosion temperature (°C)	Corrosion time (h)	Oxygen concentration (wt.%)	Bi concentration (%)	Measurement temperature (°C)	Measured thermal conductivity	Predicted thermal conductivity	Absolute deviation	Percentage deviation (%)
0.9102	0.0574	0.0014	450	3000	3.31E−04	55.5	450	25.1185	25.4787	0.3602	1.43
0.9102	0.0574	0.0014	450	3000	3.31E−04	55.5	500	25.5133	25.4194	0.0939	0.37
0.9102	0.0574	0.0014	450	1000	1.00E−06	55.5	350	25.2660	25.1174	0.1486	0.59
0.9102	0.0574	0.0014	450	1000	1.00E−06	55.5	400	25.4291	25.1156	0.3136	1.23
0.9102	0.0574	0.0014	450	1000	1.00E−06	55.5	450	25.2897	25.0989	0.1908	0.75
0.9102	0.0574	0.0014	450	1000	1.00E−06	55.5	500	25.2435	25.4073	0.0363	0.14
0.9102	0.0574	0.0014	400	1000	1.00E−06	55.5	350	26.0003	26.0292	0.0290	0.11
0.9102	0.0574	0.0014	400	1000	1.00E−06	55.5	400	26.0528	26.0274	0.0254	0.10
0.9102	0.0574	0.0014	400	1000	1.00E−06	55.5	450	26.1155	26.0566	0.0589	0.23
0.9102	0.0574	0.0014	400	1000	1.00E−06	55.5	500	25.4641	26.4058	0.0584	0.22
0.9102	0.0574	0.0014	550	1000	1.00E−06	55.5	350	25.0344	24.7934	0.2409	0.96
0.9102	0.0574	0.0014	550	1000	1.00E−06	55.5	400	25.2162	24.7934	0.4227	1.68
0.9102	0.0574	0.0014	550	1000	1.00E−06	55.5	450	24.3801	24.7402	0.6398	2.52
0.9102	0.0574	0.0014	550	1000	1.00E−06	55.5	500	24.1137	25.2342	0.5795	2.24

Fe (wt.%)	Cr (wt.%)	Ni (wt.%)	Corrosion temperature (°C)	Corrosion time (h)	Oxygen concentration (wt.%)	Bi concentration (%)	Measurement temperature (°C)	Measured thermal conductivity	Predicted thermal conductivity	Absolute deviation	Percentage deviation (%)
0.9102	0.0574	0.0014	550	2000	1.00E−06	55.5	350	24.3831	24.1716	0.2114	0.87
0.9102	0.0574	0.0014	550	2000	1.00E−06	55.5	400	24.5812	24.1716	0.4096	1.67
0.9102	0.0574	0.0014	550	2000	1.00E−06	55.5	450	24.3623	24.1566	0.2057	0.84
0.9102	0.0574	0.0014	550	2000	1.00E−06	55.5	500	24.6872	24.1265	0.5607	2.27
0.9102	0.0574	0.0014	600	4000	1.00E−06	55.5	350	22.6655	22.7640	0.0985	0.43
0.9102	0.0574	0.0014	600	4000	1.00E−06	55.5	400	22.5494	22.7640	0.2146	0.95
0.9102	0.0574	0.0014	600	4000	1.00E−06	55.5	450	21.8048	22.3054	0.5006	2.30
0.9102	0.0574	0.0014	600	4000	1.00E−06	55.5	500	21.4591	21.9928	0.5337	2.49
0.9102	0.0574	0.0014	500	2000	1.00E−06	55.5	350	21.9627	21.8251	0.1376	0.63
0.9102	0.0574	0.0014	500	2000	1.00E−06	55.5	400	21.7324	21.8251	0.0926	0.43
0.9102	0.0574	0.0014	500	2000	1.00E−06	55.5	450	21.1348	21.7501	0.6153	2.91
0.9102	0.0574	0.0014	500	2000	1.00E−06	55.5	500	20.6228	21.6222	0.9994	4.85
0.9102	0.0574	0.0014	600	2000	1.00E−06	53	350	19.0659	18.9191	0.1468	0.77
0.9102	0.0574	0.0014	600	2000	1.00E−06	53	400	18.8812	18.9191	0.0379	0.20

Fe (wt.%)	Cr (wt.%)	Ni (wt.%)	Corrosion temperature (°C)	Corrosion time (h)	Oxygen concentration (wt.%)	Bi concentration (%)	Measurement temperature (°C)	Measured thermal conductivity	Predicted thermal conductivity	Absolute deviation	Percentage deviation (%)
0.9102	0.0574	0.0014	600	2000	1.00E−06	53	450	18.2027	18.2779	0.0752	0.41
0.9102	0.0574	0.0014	600	2000	1.00E−06	53	500	17.8893	18.0696	0.1803	1.01
0.9102	0.0574	0.0014	600	2000	1.00E−06	50	350	17.6149	17.6583	0.0434	0.25
0.9102	0.0574	0.0014	600	2000	1.00E−06	50	400	17.7360	17.6583	0.0777	0.44
0.9102	0.0574	0.0014	600	2000	1.00E−06	50	450	17.1677	17.3462	0.1786	1.04
0.9102	0.0574	0.0014	600	2000	1.00E−06	50	500	16.7879	17.0777	0.2897	1.73
0.9102	0.0574	0.0014	550	2500	1.00E−06	55.5	350	22.2611	22.0712	0.1899	0.85
0.9102	0.0574	0.0014	550	2500	1.00E−06	55.5	400	22.0219	22.0712	0.0494	0.22
0.9102	0.0574	0.0014	550	2500	1.00E−06	55.5	450	21.4547	21.9992	0.5445	2.54
0.9102	0.0574	0.0014	550	2500	1.00E−06	55.5	500	21.3337	21.7096	0.3759	1.76
0.9102	0.0574	0.0014	500	3500	1.00E−08	55.5	350	20.9158	21.4140	0.4982	2.38
0.9102	0.0574	0.0014	500	3500	1.00E−08	55.5	400	20.6396	21.4140	0.7744	3.75
0.9102	0.0574	0.0014	500	3500	1.00E−08	55.5	450	19.7493	21.0896	1.3402	6.79
0.9102	0.0574	0.0014	500	3500	1.00E−08	55.5	500	19.7969	20.5560	0.7591	3.83

Fe (wt.%)	Cr (wt.%)	Ni (wt.%)	Corrosion temperature (°C)	Corrosion time (h)	Oxygen concentration (wt.%)	Bi concentration (%)	Measurement temperature (°C)	Measured thermal conductivity	Predicted thermal conductivity	Absolute deviation	Percentage deviation (%)
0.9102	0.0574	0.0014	450	3500	1.00E−06	55.5	350	20.4686	20.5823	0.1136	0.56
0.9102	0.0574	0.0014	450	3500	1.00E−06	55.5	400	20.1957	20.5823	0.3866	1.91
0.9102	0.0574	0.0014	450	3500	1.00E−06	55.5	450	19.4436	20.5823	1.1386	5.86
0.9102	0.0574	0.0014	450	3500	1.00E−06	55.5	500	19.4710	20.2391	0.7680	3.94
0.9102	0.0574	0.0014	400	3500	1.00E−06	55.5	350	20.6769	20.4330	0.2439	1.18
0.9102	0.0574	0.0014	400	3500	1.00E−06	55.5	400	20.4224	20.4330	0.0106	0.05
0.9102	0.0574	0.0014	400	3500	1.00E−06	55.5	450	19.6942	20.1587	0.4644	2.36
0.9102	0.0574	0.0014	400	3500	1.00E−06	55.5	500	19.6976	19.8154	0.1178	0.60
0.9102	0.0574	0.0014	350	4000	3.45E−05	55.5	350	25.9828	25.4195	0.5633	2.17
0.9102	0.0574	0.0014	350	4000	3.45E−05	55.5	400	25.4566	25.3889	0.0677	0.27
0.9102	0.0574	0.0014	350	4000	3.45E−05	55.5	450	24.6627	25.1334	0.4707	1.91
0.9102	0.0574	0.0014	350	4000	3.45E−05	55.5	500	24.5387	24.8632	0.3246	1.32
0.7568	0.1135	0.0694	450	4000	1.00E−08	55.5	350	16.8744	17.0185	0.1441	0.85
0.7568	0.1135	0.0694	450	4000	1.00E−08	55.5	400	17.5807	17.7618	0.1811	1.03

Fe (wt.%)	Cr (wt.%)	Ni (wt.%)	Corrosion temperature (°C)	Corrosion time (h)	Oxygen concentration (wt.%)	Bi concentration (%)	Measurement temperature (°C)	Measured thermal conductivity	Predicted thermal conductivity	Absolute deviation	Percentage deviation (%)
0.7568	0.1135	0.0694	450	4000	1.00E−08	55.5	450	19.6767	19.1073	0.5695	2.89
0.7568	0.1135	0.0694	450	4000	1.00E−08	55.5	500	19.2375	19.6839	0.4464	2.32
0.7568	0.1135	0.0694	500	4000	1.00E−08	55.5	350	17.0369	17.2173	0.1804	1.06
0.7568	0.1135	0.0694	500	4000	1.00E−08	55.5	400	17.7126	17.5789	0.1337	0.75
0.7568	0.1135	0.0694	500	4000	1.00E−08	55.5	450	19.9457	18.7251	1.2206	6.12
0.7568	0.1135	0.0694	500	4000	1.00E−08	55.5	500	19.5274	19.3115	0.2159	1.11
0.7568	0.1135	0.0694	400	2000	1.00E−06	55.5	350	17.0748	17.1909	0.1160	0.68
0.7568	0.1135	0.0694	400	2000	1.00E−06	55.5	400	17.9579	18.0616	0.1037	0.58
0.7568	0.1135	0.0694	400	2000	1.00E−06	55.5	450	19.2576	19.1541	0.1035	0.54
0.7568	0.1135	0.0694	400	2000	1.00E−06	55.5	500	19.5440	19.6917	0.1477	0.76
0.7568	0.1135	0.0694	600	3000	1.00E−06	50	350	16.9076	16.9700	0.0624	0.37
0.7568	0.1135	0.0694	600	3000	1.00E−06	50	400	17.4270	17.4631	0.0360	0.21
0.7568	0.1135	0.0694	600	3000	1.00E−06	50	450	18.6810	18.3276	0.3533	1.89
0.7568	0.1135	0.0694	600	3000	1.00E−06	50	500	18.6429	18.8656	0.2227	1.19

Fe (wt.%)	Cr (wt.%)	Ni (wt.%)	Corrosion temperature (°C)	Corrosion time (h)	Oxygen concentration (wt.%)	Bi concentration (%)	Measurement temperature (°C)	Measured thermal conductivity	Predicted thermal conductivity	Absolute deviation	Percentage deviation (%)
0.7568	0.1135	0.0694	600	1500	1.00E−08	55.5	350	15.0723	15.1849	0.1126	0.75
0.7568	0.1135	0.0694	600	1500	1.00E−08	55.5	400	15.6526	15.8099	0.1574	1.01
0.7568	0.1135	0.0694	600	1500	1.00E−08	55.5	450	16.7733	16.7015	0.0718	0.43
0.7568	0.1135	0.0694	600	1500	1.00E−08	55.5	500	16.6907	16.9176	0.2269	1.36
0.7568	0.1135	0.0694	450	5000	1.00E−08	55.5	350	16.7319	16.5599	0.1719	1.03
0.7568	0.1135	0.0694	450	5000	1.00E−08	55.5	400	17.1335	16.9803	0.1532	0.89
0.7568	0.1135	0.0694	450	5000	1.00E−08	55.5	450	17.0062	17.3184	0.3123	1.84
0.7568	0.1135	0.0694	450	5000	1.00E−08	55.5	500	18.3562	18.2293	0.1269	0.69
0.7568	0.1135	0.0694	500	5500	1.00E−08	55.5	350	15.6111	15.8017	0.1906	1.22
0.7568	0.1135	0.0694	500	5500	1.00E−08	55.5	400	16.1275	15.9436	0.1840	1.14
0.7568	0.1135	0.0694	500	5500	1.00E−08	55.5	450	15.9884	15.8096	0.1788	1.12
0.7568	0.1135	0.0694	500	5500	1.00E−08	55.5	500	16.9212	16.8905	0.0307	0.18
0.7568	0.1135	0.0694	600	8500	1.00E−08	55.5	350	16.1619	16.0936	0.0683	0.42
0.7568	0.1135	0.0694	600	8500	1.00E−08	55.5	400	16.2636	16.2876	0.0240	0.15

Fe (wt.%)	Cr (wt.%)	Ni (wt.%)	Corrosion temperature (°C)	Corrosion time (h)	Oxygen concentration (wt.%)	Bi concentration (%)	Measurement temperature (°C)	Measured thermal conductivity	Predicted thermal conductivity	Absolute deviation	Percentage deviation (%)
0.7568	0.1135	0.0694	600	8500	1.00E−08	55.5	450	15.9184	16.0439	0.1255	0.79
0.7568	0.1135	0.0694	600	8500	1.00E−08	55.5	500	16.3818	16.3775	0.0043	0.03
0.7568	0.1135	0.0694	500	7000	1.00E−06	55.5	350	14.3626	14.7967	0.4341	3.02
0.7568	0.1135	0.0694	500	7000	1.00E−06	55.5	400	14.6083	14.7038	0.0955	0.65
0.7568	0.1135	0.0694	500	7000	1.00E−06	55.5	450	14.4828	14.5662	0.0835	0.58
0.7568	0.1135	0.0694	500	7000	1.00E−06	55.5	500	15.1217	15.1216	0.0001	0.00
0.7568	0.1135	0.0694	450	9000	1.00E−06	55.5	350	15.0465	15.0952	0.0487	0.32
0.7568	0.1135	0.0694	450	9000	1.00E−06	55.5	400	15.3110	15.2193	0.0918	0.60
0.7568	0.1135	0.0694	450	9000	1.00E−06	55.5	450	15.3175	15.0077	0.3097	2.02
0.7568	0.1135	0.0694	450	9000	1.00E−06	55.5	500	15.6507	15.6033	0.0474	0.30
0.7568	0.1135	0.0694	500	1000	1.00E−08	55.5	350	14.1238	14.1593	0.0355	0.25
0.7568	0.1135	0.0694	500	1000	1.00E−08	55.5	400	14.4948	14.5887	0.0939	0.65
0.7568	0.1135	0.0694	500	1000	1.00E−08	55.5	450	14.5047	14.5773	0.0726	0.50
0.7568	0.1135	0.0694	500	1000	1.00E−08	55.5	500	14.7132	14.7700	0.0568	0.39

Fe (wt.%)	Cr (wt.%)	Ni (wt.%)	Corrosion temperature (°C)	Corrosion time (h)	Oxygen concentration (wt.%)	Bi concentration (%)	Measurement temperature (°C)	Measured thermal conductivity	Predicted thermal conductivity	Absolute deviation	Percentage deviation (%)
0.7568	0.1135	0.0694	600	3000	1.00E−06	53	350	13.7704	13.7747	0.0044	0.03
0.7568	0.1135	0.0694	600	3000	1.00E−06	53	400	13.9688	13.9862	0.0174	0.12
0.7568	0.1135	0.0694	600	3000	1.00E−06	53	450	13.9077	14.5315	0.6238	4.49
0.7568	0.1135	0.0694	600	3000	1.00E−06	53	500	14.0409	15.9061	1.8652	13.28
0.7568	0.1135	0.0694	400	3000	1.16E−04	55.5	350	17.1653	17.1503	0.0150	0.09
0.7568	0.1135	0.0694	400	3000	1.16E−04	55.5	400	17.7234	17.6884	0.0350	0.20
0.7568	0.1135	0.0694	400	3000	1.16E−04	55.5	450	17.8512	18.0855	0.2343	1.31
0.7568	0.1135	0.0694	400	3000	1.16E−04	55.5	500	18.8524	18.7843	0.0682	0.36
0.7568	0.1135	0.0694	450	3000	3.31E−04	55.5	350	17.0879	17.1077	0.0198	0.12
0.7568	0.1135	0.0694	450	3000	3.31E−04	55.5	400	17.5507	17.5991	0.0484	0.28
0.7568	0.1135	0.0694	450	3000	3.31E−04	55.5	450	17.6177	17.4591	0.1585	0.90
0.7568	0.1135	0.0694	450	3000	3.31E−04	55.5	500	18.5565	17.6968	0.8597	4.63
0.7568	0.1135	0.0694	500	4000	8.23E−04	55.5	350	15.6222	15.7205	0.0983	0.63
0.7568	0.1135	0.0694	500	4000	8.23E−04	55.5	400	15.8581	15.8708	0.0127	0.08

Fe (wt.%)	Cr (wt.%)	Ni (wt.%)	Corrosion temperature (°C)	Corrosion time (h)	Oxygen concentration (wt.%)	Bi concentration (%)	Measurement temperature (°C)	Measured thermal conductivity	Predicted thermal conductivity	Absolute deviation	Percentage deviation (%)
0.7568	0.1135	0.0694	500	4000	8.23E−04	55.5	450	15.7593	15.9522	0.1929	1.22
0.7568	0.1135	0.0694	500	4000	8.23E−04	55.5	500	16.2657	16.1648	0.1009	0.62
0.7568	0.1135	0.0694	450	1000	3.31E−04	55.5	350	15.6652	15.6319	0.0333	0.21
0.7568	0.1135	0.0694	450	1000	3.31E−04	55.5	400	16.2494	16.2710	0.0216	0.13
0.7568	0.1135	0.0694	450	1000	3.31E−04	55.5	450	16.7379	16.8148	0.0769	0.46
0.7568	0.1135	0.0694	450	1000	3.31E−04	55.5	500	17.7514	17.7279	0.0235	0.13
0.7568	0.1135	0.0694	600	2000	3.72E−03	57	350	16.4817	16.4700	0.0118	0.07
0.7568	0.1135	0.0694	600	2000	3.72E−03	57	400	16.7759	17.1203	0.3444	2.05
0.7568	0.1135	0.0694	600	2000	3.72E−03	57	450	17.1306	17.3370	0.2064	1.20
0.7568	0.1135	0.0694	600	2000	3.72E−03	57	500	17.7811	17.5578	0.2233	1.26
0.7568	0.1135	0.0694	600	2000	1.00E−06	57	350	18.0396	17.6497	0.3900	2.16
0.7568	0.1135	0.0694	600	2000	1.00E−06	57	400	18.4480	18.4218	0.0263	0.14
0.7568	0.1135	0.0694	600	2000	1.00E−06	57	450	19.0911	19.0255	0.0656	0.34
0.7568	0.1135	0.0694	600	2000	1.00E−06	57	500	19.6682	19.6096	0.0585	0.30

Fe (wt.%)	Cr (wt.%)	Ni (wt.%)	Corrosion temperature (°C)	Corrosion time (h)	Oxygen concentration (wt.%)	Bi concentration (%)	Measurement temperature (°C)	Measured thermal conductivity	Predicted thermal conductivity	Absolute deviation	Percentage deviation (%)
0.7568	0.1135	0.0694	600	2000	3.72E−03	50	350	15.9440	16.0802	0.1362	0.85
0.7568	0.1135	0.0694	600	2000	3.72E−03	50	400	17.7838	17.6790	0.1047	0.59
0.7568	0.1135	0.0694	600	2000	3.72E−03	50	450	18.1007	17.9460	0.1546	0.85
0.7568	0.1135	0.0694	600	2000	3.72E−03	50	500	18.7447	18.1668	0.5779	3.08
0.7568	0.1135	0.0694	600	2000	1.00E−08	55.5	350	17.9658	17.8756	0.0902	0.50
0.7568	0.1135	0.0694	600	2000	1.00E−08	55.5	400	19.0162	18.9472	0.0689	0.36
0.7568	0.1135	0.0694	600	2000	1.00E−08	55.5	450	19.6876	19.5079	0.1797	0.91
0.7568	0.1135	0.0694	600	2000	1.00E−08	55.5	500	20.3265	20.2928	0.0338	0.17
0.7568	0.1135	0.0694	600	2000	1.00E−08	57	350	17.1361	17.3491	0.2130	1.24
0.7568	0.1135	0.0694	600	2000	1.00E−08	57	400	18.1490	18.2651	0.1161	0.64
0.7568	0.1135	0.0694	600	2000	1.00E−08	57	450	18.7819	18.8829	0.1010	0.54
0.7568	0.1135	0.0694	600	2000	1.00E−08	57	500	19.5247	19.5575	0.0328	0.17
0.7568	0.1135	0.0694	600	3000	1.00E−08	50	350	17.4761	17.4802	0.0041	0.02
0.7568	0.1135	0.0694	600	3000	1.00E−08	50	400	18.2594	18.2651	0.0057	0.03

Fe (wt.%)	Cr (wt.%)	Ni (wt.%)	Corrosion temperature (°C)	Corrosion time (h)	Oxygen concentration (wt.%)	Bi concentration (%)	Measurement temperature (°C)	Measured thermal conductivity	Predicted thermal conductivity	Absolute deviation	Percentage deviation (%)
0.7568	0.1135	0.0694	600	3000	1.00E−08	50	450	19.0431	19.0675	0.0245	0.13
0.7568	0.1135	0.0694	600	3000	1.00E−08	50	500	19.6151	19.6521	0.0369	0.19
0.7568	0.1135	0.0694	500	3500	1.00E−06	55.5	350	16.9035	17.1887	0.2852	1.69
0.7568	0.1135	0.0694	500	3500	1.00E−06	55.5	400	17.4885	17.3655	0.1230	0.70
0.7568	0.1135	0.0694	500	3500	1.00E−06	55.5	450	18.1571	18.0235	0.1336	0.74
0.7568	0.1135	0.0694	500	3500	1.00E−06	55.5	500	18.5043	18.6316	0.1272	0.69
0.7568	0.1135	0.0694	600	5000	1.00E−08	55.5	350	16.8928	16.8969	0.0041	0.02
0.7568	0.1135	0.0694	600	5000	1.00E−08	55.5	400	17.2457	17.2907	0.0450	0.26
0.7568	0.1135	0.0694	600	5000	1.00E−08	55.5	450	17.8165	17.7894	0.0271	0.15
0.7568	0.1135	0.0694	600	5000	1.00E−08	55.5	500	18.2721	18.2823	0.0102	0.06
0.9102	0.0574	0.0014	600	1000	3.72E−03	57	350	25.4493	25.3618	0.0875	0.34
0.9102	0.0574	0.0014	600	1000	3.72E−03	57	400	24.6662	24.6941	0.0279	0.11
0.9102	0.0574	0.0014	600	1000	3.72E−03	57	450	23.1406	23.3913	0.2507	1.08
0.9102	0.0574	0.0014	600	1000	3.72E−03	57	500	23.1178	23.6612	0.5434	2.35

Fe (wt.%)	Cr (wt.%)	Ni (wt.%)	Corrosion temperature (°C)	Corrosion time (h)	Oxygen concentration (wt.%)	Bi concentration (%)	Measurement temperature (°C)	Measured thermal conductivity	Predicted thermal conductivity	Absolute deviation	Percentage deviation (%)
0.7568	0.1135	0.0694	450	2500	1.00E−06	55.5	350	17.3530	17.2221	0.1310	0.75
0.7568	0.1135	0.0694	450	2500	1.00E−06	55.5	400	18.4130	18.2864	0.1266	0.69
0.7568	0.1135	0.0694	450	2500	1.00E−06	55.5	450	19.0073	19.0296	0.0223	0.12
0.7568	0.1135	0.0694	450	2500	1.00E−06	55.5	500	19.6857	19.5579	0.1278	0.65
0.7568	0.1135	0.0694	600	1000	3.72E−03	50	350	16.4385	16.4174	0.0211	0.13
0.7568	0.1135	0.0694	600	1000	3.72E−03	50	400	17.2387	17.1817	0.0571	0.33
0.7568	0.1135	0.0694	600	1000	3.72E−03	50	450	18.7447	17.8807	0.0013	0.01
0.7568	0.1135	0.0694	600	1000	3.72E−03	50	500	19.1697	18.1506	0.0192	0.11
0.7568	0.1135	0.0694	600	2000	1.00E−06	60	350	17.2851	17.4437	0.1586	0.92
0.7568	0.1135	0.0694	600	2000	1.00E−06	60	400	18.4420	18.2848	0.1572	0.85
0.7568	0.1135	0.0694	600	2000	1.00E−06	60	450	18.8057	18.8886	0.0829	0.44
0.7568	0.1135	0.0694	600	2000	1.00E−06	60	500	19.5015	19.4727	0.0287	0.15
0.7568	0.1135	0.0694	600	1000	3.72E−03	55.5	350	16.2405	16.4050	0.1645	1.01
0.7568	0.1135	0.0694	600	1000	3.72E−03	55.5	400	16.5039	17.1005	0.1965	1.16

Fe (wt.%)	Cr (wt.%)	Ni (wt.%)	Corrosion temperature (°C)	Corrosion time (h)	Oxygen concentration (wt.%)	Bi concentration (%)	Measurement temperature (°C)	Measured thermal conductivity	Predicted thermal conductivity	Absolute deviation	Percentage deviation (%)
0.7568	0.1135	0.0694	600	1000	3.72E−03	55.5	450	16.6656	16.7803	0.0958	0.55
0.7568	0.1135	0.0694	600	1000	3.72E−03	55.5	500	17.5298	17.4340	0.1146	0.69
0.9102	0.0574	0.0014	600	2000	1.00E−06	57	350	23.1153	23.2515	0.1363	0.59
0.9102	0.0574	0.0014	600	2000	1.00E−06	57	400	23.1630	23.2515	0.0885	0.38
0.9102	0.0574	0.0014	600	2000	1.00E−06	57	450	23.4811	23.3598	0.1212	0.52
0.9102	0.0574	0.0014	600	2000	1.00E−06	57	500	23.8398	23.3142	0.5257	2.20
0.9102	0.0574	0.0014	500	3000	8.32E−04	57	350	23.7839	23.8437	0.0598	0.25
0.9102	0.0574	0.0014	500	3000	8.32E−04	57	400	24.2598	23.8407	0.4191	1.73
0.9102	0.0574	0.0014	500	3000	8.32E−04	57	450	23.1406	23.1090	0.0315	0.14
0.9102	0.0574	0.0014	500	3000	8.32E−04	57	500	20.9190	21.4114	0.4924	2.35
0.9102	0.0574	0.0014	500	3000	8.32E−04	50	350	25.1146	24.9907	0.1238	0.49
0.9102	0.0574	0.0014	500	3000	8.32E−04	50	400	24.6999	24.7989	0.0991	0.40
0.9102	0.0574	0.0014	500	3000	8.32E−04	50	450	24.2882	24.2939	0.0057	0.02
0.9102	0.0574	0.0014	500	3000	8.32E−04	50	500	24.2663	23.5628	0.7035	2.90

Fe (wt.%)	Cr (wt.%)	Ni (wt.%)	Corrosion temperature (°C)	Corrosion time (h)	Oxygen concentration (wt.%)	Bi concentration (%)	Measurement temperature (°C)	Measured thermal conductivity	Predicted thermal conductivity	Absolute deviation	Percentage deviation (%)
0.9102	0.0574	0.0014	500	3000	8.32E−04	60	350	24.5504	24.6194	0.0690	0.28
0.9102	0.0574	0.0014	500	3000	8.32E−04	60	400	24.2371	24.4784	0.2414	1.00
0.9102	0.0574	0.0014	500	3000	8.32E−04	60	450	24.0416	24.0188	0.0228	0.09
0.9102	0.0574	0.0014	500	3000	8.32E−04	60	500	23.7096	23.5095	0.2001	0.84
0.9102	0.0574	0.0014	600	7500	1.00E−06	55.5	350	25.4262	25.3476	0.0786	0.31
0.9102	0.0574	0.0014	600	7500	1.00E−06	55.5	400	25.1495	25.1583	0.0088	0.04
0.9102	0.0574	0.0014	600	7500	1.00E−06	55.5	450	25.6267	25.4794	0.1473	0.57
0.9102	0.0574	0.0014	600	7500	1.00E−06	55.5	500	25.1487	25.1598	0.0111	0.04
0.9102	0.0574	0.0014	450	2500	1.00E−06	55.5	350	21.2012	22.0103	0.8091	3.82
0.9102	0.0574	0.0014	450	2500	1.00E−06	55.5	400	21.8532	22.1912	0.3380	1.55
0.9102	0.0574	0.0014	450	2500	1.00E−06	55.5	450	21.7983	22.2197	0.4214	1.93
0.9102	0.0574	0.0014	450	2500	1.00E−06	55.5	500	21.8401	21.8949	0.0548	0.25
0.9102	0.0574	0.0014	450	4000	1.00E−08	55.5	350	23.6236	24.0598	0.4363	1.85
0.9102	0.0574	0.0014	450	4000	1.00E−08	55.5	400	24.2555	24.0779	0.1776	0.73

Fe (wt.%)	Cr (wt.%)	Ni (wt.%)	Corrosion temperature (°C)	Corrosion time (h)	Oxygen concentration (wt.%)	Bi concentration (%)	Measurement temperature (°C)	Measured thermal conductivity	Predicted thermal conductivity	Absolute deviation	Percentage deviation (%)
0.9102	0.0574	0.0014	450	4000	1.00E−08	55.5	450	24.0327	24.7562	0.7235	3.01
0.9102	0.0574	0.0014	450	4000	1.00E−08	55.5	500	24.3145	24.3297	0.0152	0.06
0.9102	0.0574	0.0014	500	3500	1.00E−06	55.5	350	23.1167	23.0409	0.0757	0.33
0.9102	0.0574	0.0014	500	3500	1.00E−06	55.5	400	23.5281	23.0409	0.4872	2.07
0.9102	0.0574	0.0014	500	3500	1.00E−06	55.5	450	23.5244	23.0409	0.4835	2.06
0.9102	0.0574	0.0014	500	3500	1.00E−06	55.5	500	23.5988	22.6988	0.8999	3.81
0.9102	0.0574	0.0014	600	3500	1.00E−08	55.5	350	26.5727	26.3370	0.2357	0.89
0.9102	0.0574	0.0014	600	3500	1.00E−08	55.5	400	26.9411	26.3370	0.6041	2.24
0.9102	0.0574	0.0014	600	3500	1.00E−08	55.5	450	26.7656	26.3370	0.4286	1.60
0.9102	0.0574	0.0014	600	3500	1.00E−08	55.5	500	25.0864	25.0333	0.0531	0.21
0.7568	0.1135	0.0694	600	3000	1.00E−08	60	350	15.5774	15.6835	0.1061	0.68
0.7568	0.1135	0.0694	600	3000	1.00E−08	60	400	16.1774	16.3081	0.1307	0.81
0.7568	0.1135	0.0694	600	3000	1.00E−08	60	450	17.0746	17.2033	0.1287	0.75
0.7568	0.1135	0.0694	600	3000	1.00E−08	60	500	17.0780	17.2543	0.1764	1.03

Fe (wt.%)	Cr (wt.%)	Ni (wt.%)	Corrosion temperature (°C)	Corrosion time (h)	Oxygen concentration (wt.%)	Bi concentration (%)	Measurement temperature (°C)	Measured thermal conductivity	Predicted thermal conductivity	Absolute deviation	Percentage deviation (%)
0.7568	0.1135	0.0694	600	3000	1.00E−06	60	350	17.0114	16.6514	0.3601	2.12
0.7568	0.1135	0.0694	600	3000	1.00E−06	60	400	17.4169	17.1765	0.2404	1.38
0.7568	0.1135	0.0694	600	3000	1.00E−06	60	450	18.0908	17.8380	0.2528	1.40
0.7568	0.1135	0.0694	600	3000	1.00E−06	60	500	18.3078	18.0829	0.2249	1.23
0.7568	0.1135	0.0694	350	4000	3.45E−05	55.5	350	17.4614	17.3666	0.0949	0.54
0.7568	0.1135	0.0694	350	4000	3.45E−05	55.5	400	17.7777	17.7016	0.0761	0.43
0.7568	0.1135	0.0694	350	4000	3.45E−05	55.5	450	18.5195	18.4986	0.0208	0.11
0.7568	0.1135	0.0694	350	4000	3.45E−05	55.5	500	19.2120	19.0114	0.2006	1.04
0.7568	0.1135	0.0694	600	3000	1.00E−06	60	350	16.2486	16.6514	0.4027	2.48
0.7568	0.1135	0.0694	600	3000	1.00E−06	60	400	17.0573	17.1765	0.1192	0.70
0.7568	0.1135	0.0694	600	3000	1.00E−06	60	450	17.4770	17.8380	0.3610	2.07
0.7568	0.1135	0.0694	600	3000	1.00E−06	60	500	17.9418	18.0829	0.1410	0.79
0.7568	0.1135	0.0694	600	2000	3.72E−03	57	350	16.7759	16.6247	0.1513	0.90
0.7568	0.1135	0.0694	600	2000	3.72E−03	57	400	17.1306	17.2283	0.0977	0.57

Fe (wt.%)	Cr (wt.%)	Ni (wt.%)	Corrosion temperature (°C)	Corrosion time (h)	Oxygen concentration (wt.%)	Bi concentration (%)	Measurement temperature (°C)	Measured thermal conductivity	Predicted thermal conductivity	Absolute deviation	Percentage deviation (%)
0.7568	0.1135	0.0694	600	2000	3.72E−03	57	450	17.7811	17.4262	0.3549	2.00
0.7568	0.1135	0.0694	600	2000	3.72E−03	57	500	16.4817	16.7171	0.2354	1.43
0.7568	0.1135	0.0694	600	5000	1.00E−08	55.5	350	16.8928	16.8969	0.0041	0.02
0.7568	0.1135	0.0694	600	5000	1.00E−08	55.5	400	17.2457	17.2907	0.0450	0.26
0.7568	0.1135	0.0694	600	5000	1.00E−08	55.5	450	17.8165	17.7894	0.0271	0.15
0.7568	0.1135	0.0694	600	5000	1.00E−08	55.5	500	18.2721	18.2823	0.0102	0.06
0.7568	0.1135	0.0694	600	1000	3.72E−03	57	350	14.4493	14.3074	0.1419	0.86
0.7568	0.1135	0.0694	600	1000	3.72E−03	57	400	14.6662	14.6584	0.0077	0.05
0.7568	0.1135	0.0694	600	1000	3.72E−03	57	450	14.4817	14.2449	0.0502	0.29
0.7568	0.1135	0.0694	600	1000	3.72E−03	57	500	15.5178	15.5080	0.0098	0.06
0.7568	0.1135	0.0694	600	2000	3.72E−03	50	350	15.9440	16.9250	0.9810	6.15
0.7568	0.1135	0.0694	600	2000	3.72E−03	50	400	17.7838	17.7595	0.0243	0.14
0.7568	0.1135	0.0694	600	2000	3.72E−03	50	450	18.1007	17.9867	0.1139	0.63
0.7568	0.1135	0.0694	600	2000	3.72E−03	50	500	18.7447	18.5913	0.1534	0.82

Fe (wt.%)	Cr (wt.%)	Ni (wt.%)	Corrosion temperature (°C)	Corrosion time (h)	Oxygen concentration (wt.%)	Bi concentration (%)	Measurement temperature (°C)	Measured thermal conductivity	Predicted thermal conductivity	Absolute deviation	Percentage deviation (%)
0.7568	0.1135	0.0694	600	2000	1.00E−08	55.5	350	17.9658	17.9620	0.0037	0.02
0.7568	0.1135	0.0694	600	2000	1.00E−08	55.5	400	19.0162	18.7104	0.3057	1.61
0.7568	0.1135	0.0694	600	2000	1.00E−08	55.5	450	19.6876	19.4122	0.2754	1.40
0.7568	0.1135	0.0694	600	2000	1.00E−08	55.5	500	20.3265	19.9240	0.4026	1.98
0.7568	0.1135	0.0694	600	3000	1.00E−08	57	350	17.1361	17.2382	0.1022	0.60
0.7568	0.1135	0.0694	600	3000	1.00E−08	57	400	18.1490	17.8460	0.3030	1.67
0.7568	0.1135	0.0694	600	3000	1.00E−08	57	450	18.7819	18.6201	0.1618	0.86
0.7568	0.1135	0.0694	600	3000	1.00E−08	57	500	19.5247	19.1748	0.3499	1.79
0.7568	0.1135	0.0694	600	3000	1.00E−08	50	350	17.4761	17.4070	0.0692	0.40
0.7568	0.1135	0.0694	600	3000	1.00E−08	50	400	18.2594	18.1459	0.1135	0.62
0.7568	0.1135	0.0694	600	3000	1.00E−08	50	450	19.0431	19.0739	0.0309	0.16
0.7568	0.1135	0.0694	600	3000	1.00E−08	50	500	19.6151	19.6034	0.0117	0.06
0.7568	0.1135	0.0694	600	3500	1.00E−08	55.5	350	16.3173	16.8354	0.5181	3.18
0.7568	0.1135	0.0694	600	3500	1.00E−08	55.5	400	17.2027	17.3220	0.1193	0.69

Fe (wt.%)	Cr (wt.%)	Ni (wt.%)	Corrosion temperature (°C)	Corrosion time (h)	Oxygen concentration (wt.%)	Bi concentration (%)	Measurement temperature (°C)	Measured thermal conductivity	Predicted thermal conductivity	Absolute deviation	Percentage deviation (%)
0.7568	0.1135	0.0694	600	3500	1.00E−08	55.5	450	17.7690	17.9869	0.2178	1.23
0.7568	0.1135	0.0694	600	3500	1.00E−08	55.5	500	18.4014	18.5406	0.1392	0.76
0.9102	0.0574	0.0014	600	1000	3.72E−03	50	350	25.4385	25.4907	0.0522	0.21
0.9102	0.0574	0.0014	600	1000	3.72E−03	50	400	25.2387	25.0986	0.1401	0.56
0.9102	0.0574	0.0014	600	1000	3.72E−03	50	450	24.2663	24.6467	0.3804	1.57
0.9102	0.0574	0.0014	600	1000	3.72E−03	50	500	24.1697	24.2398	0.0701	0.29
0.9102	0.0574	0.0014	600	1000	3.72E−03	55.5	350	25.4385	25.1908	0.2477	0.97
0.9102	0.0574	0.0014	600	1000	3.72E−03	55.5	400	24.2387	24.3245	0.0858	0.35
0.9102	0.0574	0.0014	600	1000	3.72E−03	55.5	450	23.7096	24.1346	0.4250	1.79
0.9102	0.0574	0.0014	600	1000	3.72E−03	55.5	500	23.1697	23.4073	0.2376	1.03
0.7568	0.1135	0.0694	600	2000	1.00E−06	60	350	17.2851	17.3476	0.0625	0.36
0.7568	0.1135	0.0694	600	2000	1.00E−06	60	400	18.4420	18.1514	0.2906	1.58
0.7568	0.1135	0.0694	600	2000	1.00E−06	60	450	18.8057	18.8126	0.0068	0.04
0.7568	0.1135	0.0694	600	2000	1.00E−06	60	500	19.5015	18.9731	0.5284	2.71

Fe (wt.%)	Cr (wt.%)	Ni (wt.%)	Corrosion temperature (°C)	Corrosion time (h)	Oxygen concentration (wt.%)	Bi concentration (%)	Measurement temperature (°C)	Measured thermal conductivity	Predicted thermal conductivity	Absolute deviation	Percentage deviation (%)
0.7568	0.1135	0.0694	600	2000	3.72E−03	55.5	350	16.2405	16.4050	0.1645	1.01
0.7568	0.1135	0.0694	600	2000	3.72E−03	55.5	400	16.9039	17.1005	0.1965	1.16
0.7568	0.1135	0.0694	600	2000	3.72E−03	55.5	450	17.5298	17.4340	0.0958	0.55
0.7568	0.1135	0.0694	600	2000	3.72E−03	55.5	500	16.6656	16.7803	0.1146	0.69
0.7568	0.1135	0.0694	600	2000	1.00E−06	57	350	17.5242	17.4244	0.0998	0.57
0.7568	0.1135	0.0694	600	2000	1.00E−06	57	400	17.9210	17.9982	0.0772	0.43
0.7568	0.1135	0.0694	600	2000	1.00E−06	57	450	18.5457	18.6860	0.1403	0.76
0.7568	0.1135	0.0694	600	2000	1.00E−06	57	500	19.1062	19.1996	0.0933	0.49
0.9102	0.0574	0.0014	600	3000	1.00E−08	55.5	350	24.7238	24.8519	0.1281	0.52
0.9102	0.0574	0.0014	600	3000	1.00E−08	55.5	400	24.6568	24.8519	0.1951	0.79
0.9102	0.0574	0.0014	600	3000	1.00E−08	55.5	450	24.8384	24.9602	0.1218	0.49
0.9102	0.0574	0.0014	600	3000	1.00E−08	55.5	500	24.9602	24.4592	0.5010	2.01
0.9102	0.0574	0.0014	500	4000	8.23E−04	55.5	350	24.2512	24.3182	0.0670	0.28
0.9102	0.0574	0.0014	500	4000	8.23E−04	55.5	400	24.0949	24.3226	0.2278	0.95

Fe (wt.%)	Cr (wt.%)	Ni (wt.%)	Corrosion temperature (°C)	Corrosion time (h)	Oxygen concentration (wt.%)	Bi concentration (%)	Measurement temperature (°C)	Measured thermal conductivity	Predicted thermal conductivity	Absolute deviation	Percentage deviation (%)
0.9102	0.0574	0.0014	500	4000	8.23E−04	55.5	450	24.2093	24.2911	0.0818	0.34
0.9102	0.0574	0.0014	500	4000	8.23E−04	55.5	500	24.1022	23.8314	0.2708	1.12
0.9102	0.0574	0.0014	500	2000	1.00E−08	55.5	350	24.9926	24.9080	0.0846	0.34
0.9102	0.0574	0.0014	500	2000	1.00E−08	55.5	400	25.2160	25.0067	0.2093	0.83
0.9102	0.0574	0.0014	500	2000	1.00E−08	55.5	450	25.3957	24.9607	0.4350	1.71
0.9102	0.0574	0.0014	500	2000	1.00E−08	55.5	500	24.8924	24.7999	0.0925	0.37
0.9102	0.0574	0.0014	350	5000	3.45E−05	55.5	350	25.8405	25.8770	0.0365	0.14
0.9102	0.0574	0.0014	350	5000	3.45E−05	55.5	400	25.8785	25.9445	0.0660	0.26
0.9102	0.0574	0.0014	350	5000	3.45E−05	55.5	450	26.2265	26.0176	0.2088	0.80
0.9102	0.0574	0.0014	350	5000	3.45E−05	55.5	500	25.8013	25.7673	0.0340	0.13
0.9102	0.0574	0.0014	450	5000	1.00E−06	55.5	350	24.5671	24.6102	0.0431	0.17
0.9102	0.0574	0.0014	450	5000	1.00E−06	55.5	400	23.7615	23.7480	0.0135	0.05
0.9102	0.0574	0.0014	450	5000	1.00E−06	55.5	450	22.8128	22.9873	0.1745	0.68
0.9102	0.0574	0.0014	450	5000	1.00E−06	55.5	500	22.3384	22.9205	0.5821	2.30

Fe (wt.%)	Cr (wt.%)	Ni (wt.%)	Corrosion temperature (°C)	Corrosion time (h)	Oxygen concentration (wt.%)	Bi concentration (%)	Measurement temperature (°C)	Measured thermal conductivity	Predicted thermal conductivity	Absolute deviation	Percentage deviation (%)
0.9102	0.0574	0.0014	600	3000	1.00E−06	60	350	26.8744	26.4941	0.3803	1.42
0.9102	0.0574	0.0014	600	3000	1.00E−06	60	400	26.6781	26.4941	0.1840	0.69
0.9102	0.0574	0.0014	600	3000	1.00E−06	60	450	27.2843	27.1312	0.1530	0.56
0.9102	0.0574	0.0014	600	3000	1.00E−06	60	500	26.8525	26.6524	0.2001	0.75
0.9102	0.0574	0.0014	450	2500	1.00E−08	55.5	350	25.6527	25.0922	0.5605	2.19
0.9102	0.0574	0.0014	450	2500	1.00E−08	55.5	400	25.5799	25.2980	0.2820	1.10
0.9102	0.0574	0.0014	450	2500	1.00E−08	55.5	450	25.8965	25.4514	0.4452	1.72
0.9102	0.0574	0.0014	450	2500	1.00E−08	55.5	500	25.2375	24.9392	0.2982	1.18
0.9102	0.0574	0.0014	500	3000	8.23E−04	50	350	25.1146	24.9907	0.1238	0.49
0.9102	0.0574	0.0014	500	3000	8.23E−04	50	400	24.6999	24.7989	0.0991	0.40
0.9102	0.0574	0.0014	500	3000	8.23E−04	50	450	24.2882	24.4370	0.1488	0.61
0.9102	0.0574	0.0014	500	3000	8.23E−04	50	500	24.2663	23.9592	0.3070	1.27
0.9102	0.0574	0.0014	500	3000	8.23E−04	60	350	24.5504	24.5226	0.0278	0.11
0.9102	0.0574	0.0014	500	3000	8.23E−04	60	400	24.2371	24.3439	0.1068	0.44

Fe (wt.%)	Cr (wt.%)	Ni (wt.%)	Corrosion temperature (°C)	Corrosion time (h)	Oxygen concentration (wt.%)	Bi concentration (%)	Measurement temperature (°C)	Measured thermal conductivity	Predicted thermal conductivity	Absolute deviation	Percentage deviation (%)
0.9102	0.0574	0.0014	500	3000	8.23E−04	60	450	24.0416	23.9810	0.0606	0.25
0.9102	0.0574	0.0014	500	3000	8.23E−04	60	500	23.7096	23.6023	0.1073	0.45
0.9102	0.0574	0.0014	600	7500	1.00E−06	55.5	350	25.4262	25.3476	0.0786	0.31
0.9102	0.0574	0.0014	600	7500	1.00E−06	55.5	400	25.1495	25.1583	0.0088	0.04
0.9102	0.0574	0.0014	600	7500	1.00E−06	55.5	450	25.6267	25.4794	0.1473	0.57
0.9102	0.0574	0.0014	600	7500	1.00E−06	55.5	500	25.1487	25.1598	0.0111	0.04
0.9102	0.0574	0.0014	600	3500	1.00E−08	55.5	350	26.5727	26.3370	0.2357	0.89
0.9102	0.0574	0.0014	600	3500	1.00E−08	55.5	400	26.9411	26.3370	0.6041	2.24
0.9102	0.0574	0.0014	600	3500	1.00E−08	55.5	450	26.7656	26.3370	0.4286	1.60
0.9102	0.0574	0.0014	600	3500	1.00E−08	55.5	500	25.0864	25.0333	0.0531	0.21
0.9102	0.0574	0.0014	450	9000	1.00E−06	55.5	350	26.3691	26.1662	0.2029	0.77
0.9102	0.0574	0.0014	450	9000	1.00E−06	55.5	400	26.0507	26.0638	0.0130	0.05
0.9102	0.0574	0.0014	450	9000	1.00E−06	55.5	450	25.0723	25.0995	0.2047	0.78
0.9102	0.0574	0.0014	450	9000	1.00E−06	55.5	500	25.0453	25.0128	0.2325	0.89

Table S2

Supplementary Validation Data

Sample name	Fe (wt.%)	Cr (wt.%)	Ni (wt.%)	Corrosion temperature (°C)	Corrosion time (h)	Measurement temperature (°C)	Oxygen concentration (wt.%)	Bi concentration (%)	Measured thermal conductivity	Predicted thermal conductivity	Percentage deviation (%)
T-400-3000-B	0.9102	0.0574	0.0014	400	3000	350	0.000116	55.5	26.6355	24.1375	9.38
T-400-3000-B	0.9102	0.0574	0.0014	400	3000	400	0.000116	55.5	26.4599	24.1375	8.78
T-400-3000-B	0.9102	0.0574	0.0014	400	3000	450	0.000116	55.5	26.4219	24.1375	8.65
T-400-3000-B	0.9102	0.0574	0.0014	400	3000	500	0.000116	55.5	26.7331	24.1375	9.71
T-600-2000-C-Bi57	0.9102	0.0574	0.0014	600	2000	350	1.00E-06	57	23.7951	23.0260	3.23
T-600-2000-C-Bi57	0.9102	0.0574	0.0014	600	2000	400	1.00E-06	57	23.8443	23.0260	3.43
T-600-2000-C-Bi57	0.9102	0.0574	0.0014	600	2000	450	1.00E-06	57	24.9601	23.0260	7.75
T-600-2000-C-Bi57	0.9102	0.0574	0.0014	600	2000	500	1.00E-06	57	24.5410	23.0260	6.17
T-500-3000-B-Bi57	0.9102	0.0574	0.0014	500	3000	350	0.000823	57	24.2388	24.1038	0.56

Sample name	Fe (wt.%)	Cr (wt.%)	Ni (wt.%)	Corrosion temperature (°C)	Corrosion time (h)	Measurement temperature (°C)	Oxygen concentration (wt.%)	Bi concentration (%)	Measured thermal conductivity	Predicted thermal conductivity	Percentage deviation (%)
T-500-3000-B-Bi57	0.9102	0.0574	0.0014	500	3000	400	0.000823	57	23.7839	24.1038	1.35
T-500-3000-B-Bi57	0.9102	0.0574	0.0014	500	3000	450	0.000823	57	24.2598	24.1038	0.64
T-500-3000-B-Bi57	0.9102	0.0574	0.0014	500	3000	500	0.000823	57	23.1406	24.1038	4.16
T-450-2500-C	0.9102	0.0574	0.0014	450	2500	350	1.00E-06	55.5	22.4461	23.0260	2.58
T-450-2500-C	0.9102	0.0574	0.0014	450	2500	400	1.00E-06	55.5	23.1364	23.0260	0.48
T-450-2500-C	0.9102	0.0574	0.0014	450	2500	450	1.00E-06	55.5	23.0783	23.0260	0.23
T-450-2500-C	0.9102	0.0574	0.0014	450	2500	500	1.00E-06	55.5	23.1225	23.0260	0.42
T-450-4000-P	0.9102	0.0574	0.0014	450	4000	350	1.00E-08	55.5	23.6236	23.6612	0.16
T-450-4000-P	0.9102	0.0574	0.0014	450	4000	400	1.00E-08	55.5	24.2555	23.6612	2.45
T-450-4000-P	0.9102	0.0574	0.0014	450	4000	450	1.00E-08	55.5	24.0327	23.6612	1.55
T-450-4000-P	0.9102	0.0574	0.0014	450	4000	500	1.00E-08	55.5	24.3145	23.6612	2.69
T-500-3500-C	0.9102	0.0574	0.0014	500	3500	350	1.00E-06	55.5	23.1167	23.0260	0.39
T-500-3500-C	0.9102	0.0574	0.0014	500	3500	400	1.00E-06	55.5	23.5281	23.0260	2.13

Sample name	Fe (wt.%)	Cr (wt.%)	Ni (wt.%)	Corrosion temperature (°C)	Corrosion time (h)	Measurement temperature (°C)	Oxygen concentration (wt.%)	Bi concentration (%)	Measured thermal conductivity	Predicted thermal conductivity	Percentage deviation (%)
T-500-3500-C	0.9102	0.0574	0.0014	500	3500	450	1.00E-06	55.5	23.5244	23.0260	2.12
T-500-3500-C	0.9102	0.0574	0.0014	500	3500	500	1.00E-06	55.5	23.5988	23.0260	2.43
3-600-3000-P	0.7568	0.1135	0.0694	600	3000	350	1.00E-08	55.5	16.7852	17.2488	2.76
3-600-3000-P	0.7568	0.1135	0.0694	600	3000	400	1.00E-08	55.5	17.6747	17.5524	0.69
3-600-3000-P	0.7568	0.1135	0.0694	600	3000	450	1.00E-08	55.5	18.4054	17.9704	2.36
3-600-3000-P	0.7568	0.1135	0.0694	600	3000	500	1.00E-08	55.5	19.2148	18.0258	6.19
3-450-4000-B	0.7568	0.1135	0.0694	450	4000	350	0.000331	55.5	17.0908	17.2262	0.79
3-450-4000-B	0.7568	0.1135	0.0694	450	4000	400	0.000331	55.5	17.6022	17.5298	0.41
3-450-4000-B	0.7568	0.1135	0.0694	450	4000	450	0.000331	55.5	18.3280	17.9478	2.07
3-450-4000-B	0.7568	0.1135	0.0694	450	4000	500	0.000331	55.5	18.9449	18.0032	4.97
3-600-2000-B-Bi53	0.7568	0.1135	0.0694	600	2000	350	0.00372	53	16.8510	17.2262	2.23
3-600-2000-B-Bi53	0.7568	0.1135	0.0694	600	2000	400	0.00372	53	17.7222	17.5298	1.09
3-600-2000-B-Bi53	0.7568	0.1135	0.0694	600	2000	450	0.00372	53	18.3995	17.9478	2.46

Sample name	Fe (wt.%)	Cr (wt.%)	Ni (wt.%)	Corrosion temperature (°C)	Corrosion time (h)	Measurement temperature (°C)	Oxygen concentration (wt.%)	Bi concentration (%)	Measured thermal conductivity	Predicted thermal conductivity	Percentage deviation (%)
3-600-2000-B-Bi53	0.7568	0.1135	0.0694	600	2000	500	0.00372	53	18.4165	18.0032	2.24
3-500-3500-C	0.7568	0.1135	0.0694	500	3500	350	1.00E-06	55.5	16.9035	17.2262	1.91
3-500-3500-C	0.7568	0.1135	0.0694	500	3500	400	1.00E-06	55.5	17.4885	17.5298	0.24
3-500-3500-C	0.7568	0.1135	0.0694	500	3500	450	1.00E-06	55.5	18.1571	17.9478	1.15
3-500-3500-C	0.7568	0.1135	0.0694	500	3500	500	1.00E-06	55.5	18.5043	18.0032	2.71
3-450-2500-C	0.7568	0.1135	0.0694	450	2500	350	1.00E-06	55.5	16.3906	17.2262	5.10
3-450-2500-C	0.7568	0.1135	0.0694	450	2500	400	1.00E-06	55.5	17.3918	17.5298	0.79
3-450-2500-C	0.7568	0.1135	0.0694	450	2500	450	1.00E-06	55.5	17.9531	17.9478	0.03
3-450-2500-C	0.7568	0.1135	0.0694	450	2500	500	1.00E-06	55.5	18.5939	18.0032	3.18
